# Human visual cortex is organized along two genetically opposed hierarchical gradients with unique developmental and evolutionary origins

**DOI:** 10.1371/journal.pbio.3000362

**Published:** 2019-07-03

**Authors:** Jesse Gomez, Zonglei Zhen, Kevin S. Weiner

**Affiliations:** 1 Helen Wills Neuroscience Institute, University of California Berkeley, Berkeley, California, United States of America; 2 Psychology Department, University of California Berkeley, Berkeley, California, United States of America; 3 Beijing Key Laboratory of Applied Experimental Psychology, Beijing Normal University, Beijing, China; 4 Faculty of Psychology, Beijing Normal University, Beijing, China; UT Southwestern Medical Center, UNITED STATES

## Abstract

Human visual cortex is organized with striking consistency across individuals. While recent findings demonstrate an unexpected coupling between functional and cytoarchitectonic regions relative to the folding of human visual cortex, a unifying principle linking these anatomical and functional features of the cortex remains elusive. To fill this gap in knowledge, we combined independent and ground truth measurements of cytoarchitectonic regions and genetic tissue characterization within human occipitotemporal cortex. Using a data-driven approach, we examined whether differential gene expression among cytoarchitectonic areas could contribute to the arealization of occipitotemporal cortex into a hierarchy based on transcriptomics. This approach revealed two opposing gene expression gradients: one that contains a series of genes with expression magnitudes that ascend from posterior (e.g., areas human occipital [hOc]1, hOc2, hOc3, etc.) to anterior cytoarchitectonic areas (e.g., areas fusiform gyrus [FG]1–FG4) and another that contains a separate series of genes that show a descending gradient from posterior to anterior areas. Using data from the living human brain, we show that each of these gradients correlates strongly with variations in measures related to either thickness or myelination of cortex, respectively. We further reveal that these genetic gradients emerge along unique trajectories in human development: the ascending gradient is present at 10–12 gestational weeks, while the descending gradient emerges later (19–24 gestational weeks). Interestingly, it is not until early childhood (before 5 years of age) that the two expression gradients achieve their adult-like mean expression values. Additional analyses in nonhuman primates (NHPs) reveal that homologous genes do not generate the same ascending and descending expression gradients as in humans. We discuss these findings relative to previously proposed hierarchies based on functional and cytoarchitectonic features of visual cortex. Altogether, these findings bridge macroscopic features of human cytoarchitectonic areas in visual cortex with microscopic features of cellular organization and genetic expression, which, despite the complexity of this multiscale correspondence, can be described by a sparse subset (approximately 200) of genes. These findings help pinpoint the genes contributing to healthy cortical development and explicate the cortical biology distinguishing humans from other primates, as well as establishing essential groundwork for understanding future work linking genetic mutations with the function and development of the human brain.

## Abstract 2

The expression of a sparse subset of human genes forms two opposed gradients that capture the processing hierarchy of visual cortex; these transcription gradients emerge at different points during human development and distinguish human from nonhuman primates.

## Introduction

One of the most reproducible findings in biology and neuroscience is the parcellation of mammalian visual cortex into areas, which are commonly considered as different stages of a processing hierarchy [1–3]. Indeed, despite debates regarding the exact number and definition of areas, as well as the serial or nonserial nature of computations among different regions, researchers acknowledge the existence of early (e.g., striate cortex, or V1), middle (e.g., areas in extrastriate cortex), and late (e.g., areas in inferior or ventral, temporal, and lateral occipitotemporal cortices) processing stages [1–3]. In humans, visual cortex has been parcellated with a multitude of methods in both living brains and postmortem tissue. E.g., areas have been identified in living brains using anatomical and functional magnetic resonance imaging (fMRI) [2–6], as well as in postmortem brains based on cytoarchitecture [7–13], myeloarchitecture [14–16], and receptor architecture [17–19]. Interestingly, and contrary to classic findings [20–23], recent research in human postmortem brains indicates a tight correspondence between cellular transitions of cytoarchitectonic areas and cortical folding throughout visual cortex [13,24–26]. Similarly, recent research also indicates a tight correspondence between folding and functional regions across visual cortex in what are considered functionally defined early [27], middle [28–30], and late [24,25,31] visual processing stages. In terms of the latter, this striking consistency is conserved across development [31–35] and is causally implicated in different aspects of visual perception [36–40]. Given this tight relationship among cellular organization, functional organization, cortical folding, and perception, the parcellation of areas within human visual cortex is an ideal test bed to ask a fundamental, yet unanswered, question in neuroscience: Are there organizational principles by which brain function and structure are linked that result in the shared brain organization and behaviors across individuals?

When faced with this question, one might quickly intuit that a feasible underlying answer is genetic in nature. Given recent work from Burt and colleagues [41] showing that genetics play a role in the broad division of cortex, it seems likely that genetics may also play a role in determining the arealization of networks within a cortical expanse such as human visual cortex. While measuring the expression of genes across discrete regions of cortex in vivo is infeasible, recent advances in transcriptomics and brain mapping have resulted in public databases detailing the transcriptome across the human cortical surface [42,43]. Furthermore, previous work from our group has provided multimodal solutions for linking different types of data to one another across spatial scales—e.g., linking functional regions at a millimeter scale in individual living human brains to cytoarchitectonic organization at a micron scale in individual postmortem brains [26,31,44]. Thus, by applying this multimodal approach to gene transcription and cytoarchitectonic data, we have the unprecedented opportunity to test how gene transcription plays a role in the arealization of human visual cortex.

To fill this gap in knowledge, we employed the Allen Human Brain Atlas (AHBA), which is a transcriptomic analysis of cortical tissue sampled throughout the neocortex in six brains, resulting in cortical surface-based maps of the expression magnitude of over 20,000 genes. Expression maps were aligned to a common anatomical space where they could be averaged across subjects and hemispheres (see Materials and Methods). To examine what role genes play in the parcellation of human visual cortex, we aligned 13 cytoarchitectonic regions of interest (cROIs) from 20 postmortem hemispheres (maximum probability maps from the Jülich atlas [7–9,11–13]: https://JüBrain.fz-juelich.de) to the same anatomical surface. By isolating those genes with the most significant differential expression across the 13 cROIs, we found that the cytoarchitectonic divisions of human visual cortex are characterized by two opposed genetic expression gradients: one that contains a series of genes with expression magnitudes that ascend from posterior (e.g., areas in human occipital cortex [hOc]1, hOc2, hOc3, etc.) to anterior cytoarchitectonic areas (e.g., areas in the human fusiform gyrus [FG]1–FG4) and another that contains a separate series of genes that show a descending gradient from posterior to anterior areas. We replicate these findings in a separate data set while providing developmental and evolutionary insight into these gradients by leveraging additional transcriptome measurements from developmental (e.g., gestational to 60 years of age) and macaque data sets. Repeating our analyses with these additional data reveals that the ascending and descending genetic gradients emerge at different points of human development (10–12 versus 19–24 gestational weeks) and that homologous genes in nonhuman primates (NHPs) do not generate the same ascending and descending expression gradients across areas as identified in humans. These findings a) identify a novel, to our knowledge, transcriptomic hierarchy of human visual cortex, b) empirically support the hypothesis that the arealization of visual cortex is tightly coupled with opposed genetic expression gradients, and c) begin to establish a biological model that links measurements at the levels of genes and cellular organization with the macroscopic features of human visual cortex. Together, these results further our understanding of the role that genes play in the differentiation of computational stages of the visual processing hierarchy.

## Results

### Opposing transcriptomic gradients contribute to the arealization of human visual cortex

**Fig 1:**
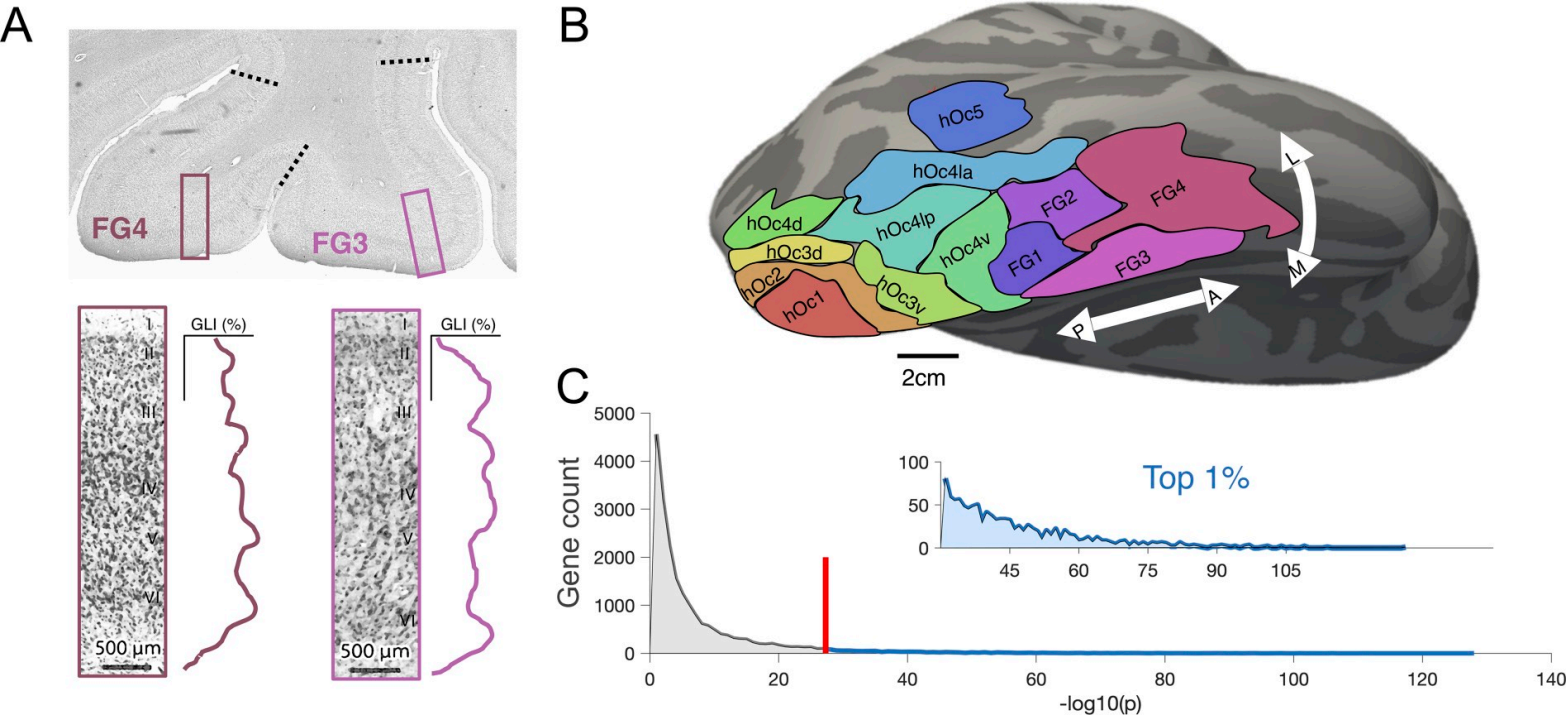
Gene selection within cytoarchitectonic neighborhoods of visual cortex. (A) A schematized version of how cytoarchitectonic areas were defined using an observer-independent algorithm in previously published work (please see [7, 78] for quantification details). Example windows taken from histological stains of human visual cortex, detailing the GLI along the cortical depth. GLIs were measured within these windows that slid along the cortex, and boundaries (black-dotted line) were drawn when GLI profiles changed significantly along several dimensions. The image of the histological section was provided by Evgeniya Kirilina. GLI profiles adapted from [63]. (B) The 13 cROIs delineated on the FreeSurfer cortical average surface. ROIs can be found at: http://www.fz-juelich.de/JüBrain/EN/_node.html. (C) In order to select genes for further analyses, we ran a 1-way ANOVA with cROI group as a factor (Materials and Methods). Histogram of the resulting *p*-values (negative log-transformed) from the ANOVA shown in gray and blue. Red line denotes the top approximately 1% (200 genes; beyond the red line and shown in blue) of genes for further analyses. Inset illustrates a zoomed-in histogram of the top 1% of genes. See S2 Fig for alternative, PCA-based expression characterization. ANOVA, analysis of variance; cROI, cytoarchitectonic region of interest; FG, fusiform gyrus; GLI, gray-level index hOc, human occipital; PCA, principal component analysis.

To identify those genes that contribute to the areal differentiation of human ventral and lateral occipitotemporal cortices, we assessed gene expression profiles that were significantly different across cROIs. In brief, cROIs were defined previously [7–13] in individual postmortem brains using the cell-density profile across layers of cortex (Fig 1A). Transition points from one cytoarchitectonic region to another were defined by an observer-independent algorithm that was blind to cortical folding. Once cROIs were defined in individual participants, they were projected from 2D histological slices to 3D cortical surface reconstructions, allowing all cROIs from each individual to be aligned using cortex-based alignment (CBA) to a common space. Maximum probability ROIs describing the most consistent location of a given cROI across postmortem brains are shown in Fig 1B. Within each cROI, there were multiple tissue samples (sample numbers by cROI shown in S1 Fig), each of which was submitted to DNA microarray analysis to quantify the expression of 20,737 genes. For each gene, we tested (e.g., with an analysis of variance [ANOVA] as done in recent work also linking cytoarchitectonic and transcriptomic data, but in the frontal lobe [45]) whether the expression levels from tissue samples were significantly different across cROIs. To equalize tissue sample numbers per ROI for the ANOVA, we grouped the cROIs into four groups (S1 Fig). A histogram (Fig 1C) of resultant *p*-values (negative log-transformed) for each gene revealed that over 50% of quantified genes are not significantly different in their expression levels across cROIs (all *p*-values > 0.05). In order to target those genes that most strongly contribute to the areal differentiation of visual cortex and to correct for multiple comparisons, we restricted our further analyses to only the top 1% (*n* = 200) of genes that were most differentially expressed. We also discuss an alternative gene selection method at the end of this section that replicated our results (S2 Fig).

**Fig 2:**
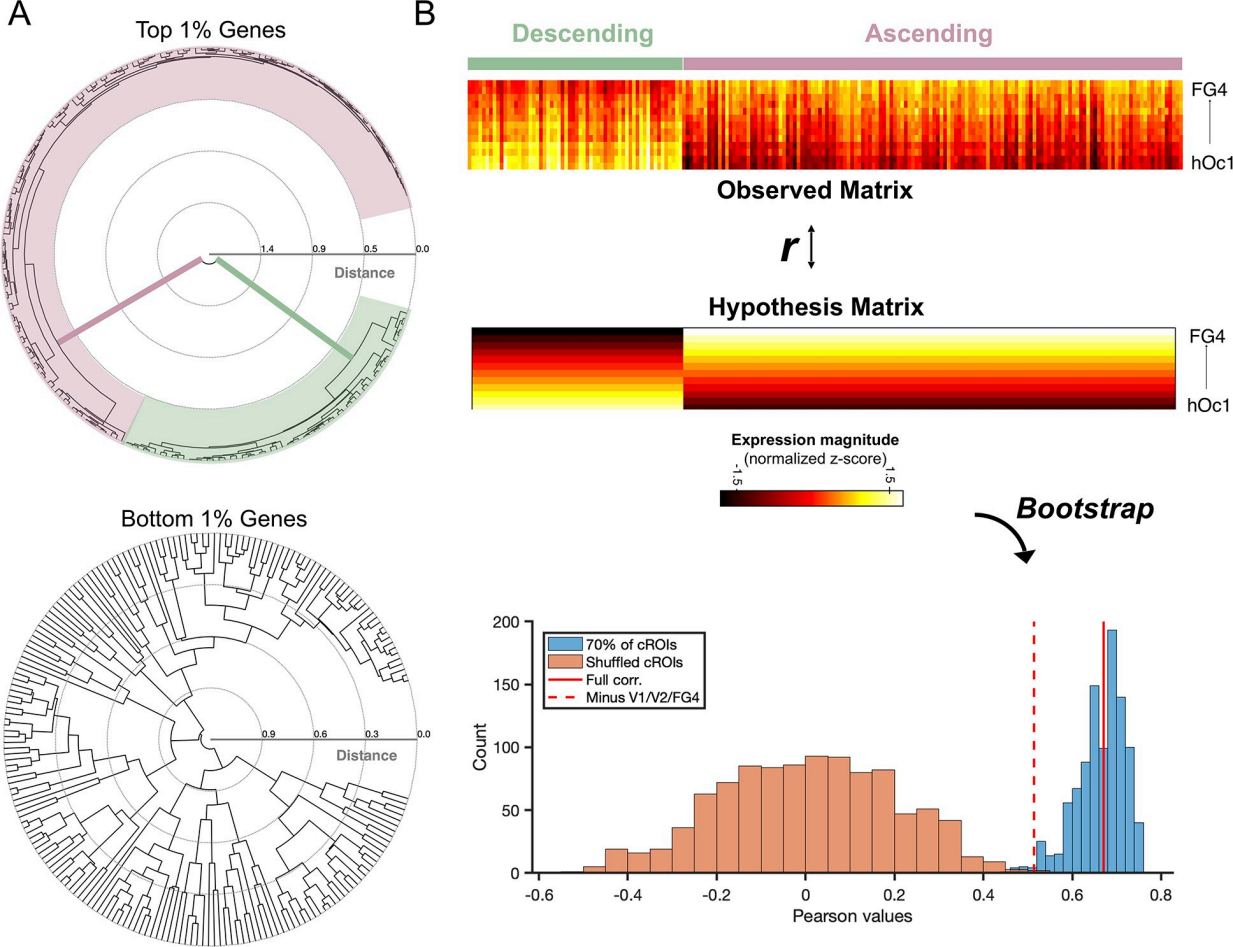
Binary clustering of the top 1% of genes reveals opposed expression gradients in human visual cortex. (A) Dendrograms showing the algorithmic clustering of the top and bottom 1% of genes. Top 1% of genes cluster predominantly into two groups at the highest level, colored here in pink and green. (B) A 13 × 200 matrix in which each row is an ROI arranged according to numerical order of the area labels (e.g., hOc1, hOc2, the hOc3 cluster, the hOc4 cluster, hOc5, and then the FG cluster) and each column is a gene. Two distinct expression groups are visible: 1) a group with a descending gradient (green) in which genes have the highest expression in posterior visual areas (e.g., hOc1, hOc2, etc.) and 2) an ascending gradient (pink) in which genes show increasing expression levels into ventral temporal visual areas (e.g., FG1, FG2, etc.). Expression levels are normalized to the maximum expression level (reads per kilobase million) across all tissue samples, and warmer colors indicate higher expression levels. To evaluate the significance of the expression matrix, we produced an equally sized hypothesis matrix in which a) columns corresponded to genes and b) column values linearly decreased or increased according to gene gradient membership. A bootstrapping (*n* = 1,000) approach was taken, shuffling columns of expression to produce a null distribution (orange histogram). Repeating the bootstrap with a subset of cROIs (75% of rows) results in the blue histogram. Solid red line marks the correlation between the original expression matrix and the hypothesis matrix (Pearson r = 0.67, *p* < 0.001). Red-dotted line denotes the correlation between the expression and hypothesis matrix when hOc1, hOc2, and FG4 were excluded, which is statistically significant, even when evaluated against the orange noise distribution (*p* = 0.002). Transcription data can be downloaded from the Allen Institute: http://brain-map.org/. cROI, cytoarchitectonic region of interest; FG, fusiform gyrus; hOc, human occipital.

To gain a better understanding of the gene expression patterns across cytoarchitectonic regions, the top 200 genes were submitted to an agglomerative hierarchical clustering algorithm (see Materials and Methods for details). Surprisingly, genes clustered predominately into two groups at the highest level, whose branch distance dwarfed all other clustering levels (Fig 2A, top). As a comparative analysis, we repeated this clustering algorithm on the bottom 200 genes (smallest 200 *p*-values from the gene histogram). This analysis underscores the striking size of the binary grouping that was unique to the top 200 genes because the bottom 200 genes revealed a much more homogenous branching pattern across cluster levels (Fig 2A, bottom). We next produced a raster plot of gene expression magnitude (z-score normalized by gene) to examine the expression patterns driving the split between the top genes. Strikingly, the two gene clusters are organized into two groups defined by opposed expression gradients. In Fig 2B, the areas (rows) are arranged according to numerical order of the area labels (e.g., hOc1, hOc2, the hOc3 cluster, the hOc4 cluster, hOc5, and then the FG cluster). Columns are organized broadly by group membership (e.g., ascending or descending gradient) in which individual gene ordering is otherwise random. One group forms a descending gradient whose composite genes express most highly in hOc1 (e.g., striate cortex) and decrease as one ascends to hOc2, hOc3, and so forth until eventually reaching the minimum level in FG4. The other group forms an ascending gradient with increasing expression levels from hOc1 to FG4. Proportionally, there are a greater number of genes contributing to the ascending gradient (two-thirds) compared to the descending gradient (one-third). For a list of the ascending and descending genes, we ask the reader to refer to S1 Table.

To evaluate the statistical significance of the visible gradients within this expression matrix, we produced a hypothesis matrix of equal size with columns that corresponded to each gene and rows whose values either linearly increased or decreased according to the gene gradient membership (Fig 2B). When assessing these comparisons, we also performed a bootstrapping procedure in which we calculated the correlation between a) the empirically observed expression matrix and b) the hypothesis matrix while shuffling the row order of the original expression matrix on each bootstrap. This approach produced a noise distribution that reveals that the correlation of the expression matrix with the hypothesis matrix is significant (r = 0.67, *p* < 0.001), even when subsampling 75% of the cROIs or removing the extrema of the cROIs (hOc1, hOc2, and FG4; *p* = 0.002; Fig 2B, bottom). It is worth emphasizing that these results are not dependent on how we select and rank the genes. E.g., the present approach of selecting genes by *p*-values (as described in Fig 1) does not take into consideration the effect size of expression differences between regions. Nevertheless, when submitting all gene expression profiles (not just the top 1%) of the 13 cROIs to a principal component analysis (PCA), we replicate our results, whereby the first component captures ascending and descending expression patterns within the most differentially expressed genes (S2 Fig). Taken together, opposing transcriptomic gradients contribute to the arealization of human visual cortex, and the identification of these gradients is not dependent on methods used for gene selection.

### Clustering based on ascending and descending gradients produces an ordering of cytoarchitectonic regions reflective of a novel transcriptomic hierarchy in human visual cortex

**Fig 3:**
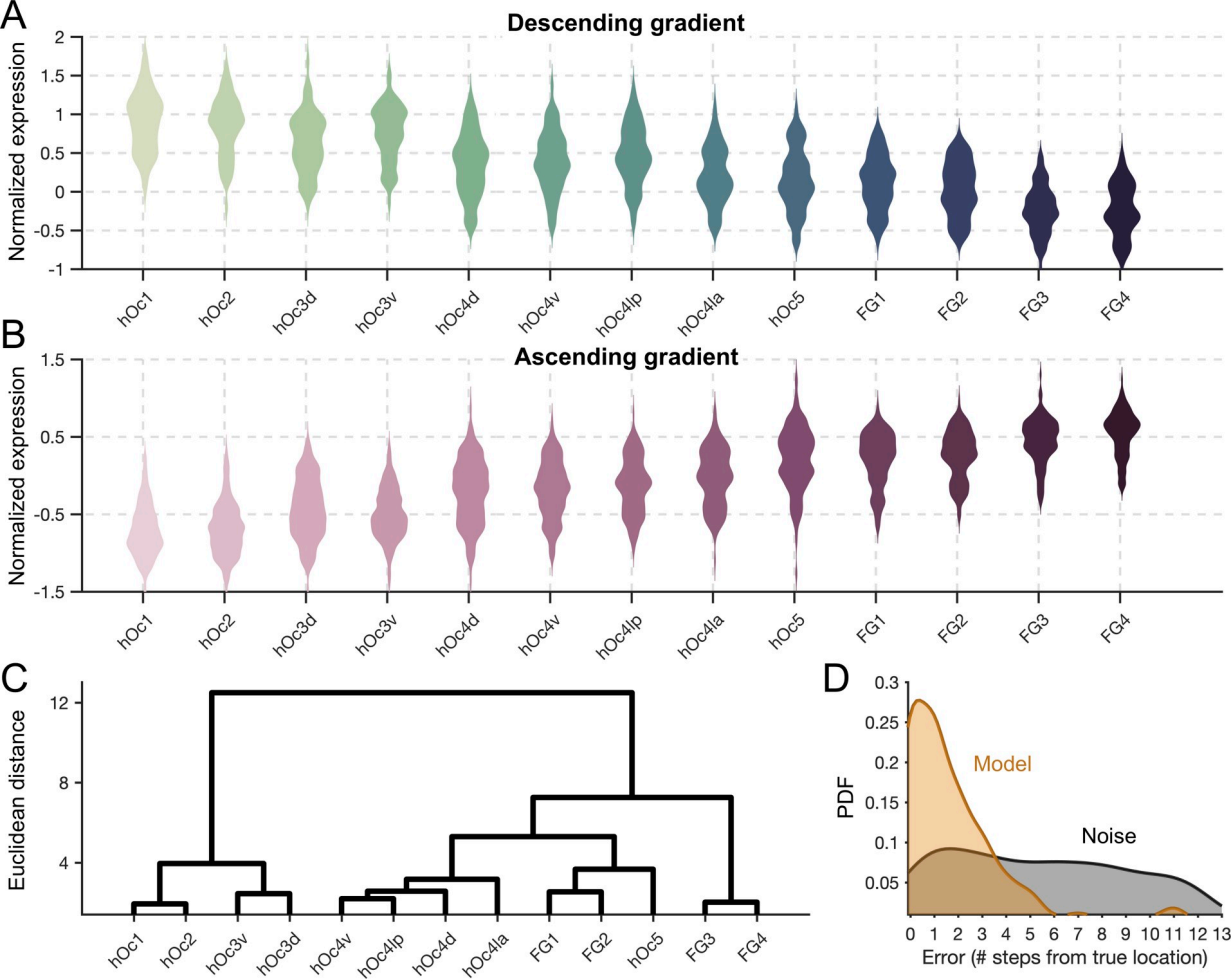
Average expression levels of the ascending and descending genetic gradients produce a transcriptomic processing hierarchy in visual cortex. (A) Gene expression levels (z-score normalized) shown across cytoarchitectonic regions for descending-gradient genes. Color shade denotes numerical order of the area labels (e.g., hOc1, hOc2, the hOc3 cluster, the hOc4 cluster, hOc5, and then the FG cluster). (B) Same as A, but for ascending gradient gene expressions. (C) Dendrogram produced from an algorithm that clustered the cROIs based on expression levels within the two gene gradients. The ordering of cROIs along the x-axis in the dendrogram is meaningful because it represents the distance from hOc1 at the level of dendrogram leaves. Consequently, this dendrogram has rooted leaves, and the clusters at this lowest level are not rotatable (Materials and Methods). (D) Distributions of the error values resulting from the model predicting a given tissue sample’s anatomical origin from its expression pattern of the top 200 genes. Goldenrod distribution shows error from the model, while gray distribution reflects error from the noise control in which gene labels were shuffled. The y-axis is probability density function, and x-axis values are the number of hierarchical steps (e.g., V1 to V2 is one step). Transcription data can be downloaded from the Allen Institute website: http://brain-map.org/. cROI, cytoarchitectonic region of interest; FG, fusiform gyrus; hOc, human occipital; hOc d, hOc dorsal; hOc la, hOc lateral anterior; hOc lp, hOc lateral posterior; hOc v, hOc ventral.

To further explore the relationship between these two opposing gene gradients and the arealization of visual cortex, we implemented a twofold procedure. First, we averaged expression patterns within the descending (Fig 3A) and ascending (Fig 3B) gradient gene clusters. Second, we submitted the expression patterns of the top 200 genes (averaged across all tissue samples within a cROI) from the 13 cROIs to an agglomerative clustering algorithm. Before clustering, cROIs were ordered according to the Euclidean distance of their expression profile from that of hOc1, such that the leaf ordering of the x-axis in the resulting dendrogram meaningfully reflects inter-regional distances from hOc1. Branches, of course, were not allowed to cross in the final clustering, so the final leaf ordering is largely, but not strictly, reflective of each cROI’s expression distance from hOc1. As such, this rooted-leaf dendrogram (Fig 3C) has clusters that cannot be rotated at the lowest level as in other dendrograms.

To our knowledge, this 2-fold approach produces a transcriptomic hierarchical ordering of the modern cytoarchitectonic parcellation of human visual cortex for the first time. At its highest level, this approach produces a dendrogram that clusters early (hOc1, hOc2, hOc 3 ventral [hOc3v], and hOc 3 dorsal [hOc3d]) from mid and late visual areas (hOc4v, hOc4d, hOc 4 lateral posterior [hOc4lp], hOc 4 lateral anterior [hOc4la], hOc5, FG1, FG2, FG3, and FG4). At the next cluster level, hOc1 and hOc2 occupy a cluster separate from that of hOc3d and hOc3v. The next closest cluster contains the hOc4 regions, followed by hOc5 and FG1 and FG2. The last cluster, and the most distant from hOc1, contains FG3 and FG4. From this point on, cROIs will be ordered according to this transcriptomic hierarchy.

To complement our previous analyses and to formally test whether genetic transcription contributes to the hierarchical distinction of regions within human visual cortex, we produced a model in which we attempted to guess the anatomical origin of a given tissue sample from the AHBA database simply from its expression profile of the top 200 genes. If genetic transcription largely contributes to the hierarchical distinction of cortical regions, then we should be able to identify a given tissue sample’s anatomical origin within human visual cortex from its transcriptional fingerprint. To test this hypothesis, we used a leave-one-out cross-validation procedure in which a given tissue sample was extracted (test sample), and from the remaining tissue samples, the expression fingerprint of each cROI was produced by averaging the expression magnitude of the top 200 genes across tissue samples that resided within a given cROI. A winner-take-all approach was used to identify the maximum correlation between the test sample’s expression pattern and the pattern of each cROI. Error was calculated as the distance (number of hierarchical steps) from the test sample’s true anatomical origin and the cROI location of its peak correlation from the model. This was repeated *n* = 215 times for all of the tissue samples. Results demonstrate that the model performs significantly above chance (assessed by bootstrapping, *n* = 1,000, the model with shuffled gene labels), with a median distance error of only 1 hierarchical step (Fig 3D).

While previous research has shown that gene expression in humans [43,46] and mice [47,48] is related to macroanatomical proximity in cortex, we emphasize that the clustering within the dendrogram in Fig 3C is not just reflective of macroanatomical proximity. E.g., hOc3d and hOc3v are within the same subcluster, and yet they are located centimeters apart in cortex. Likewise, hOc4v is located within the posterior transverse collateral sulcus in ventral occipitotemporal cortex [26], while hOc4d is located near the transverse occipital sulcus on the lateral surface of the brain [10], and yet both areas are positioned in the same subcluster. We thus sought to formally clarify what role macroanatomical proximity plays in inter-regional differences in gene expression. We posited that the transcriptional gradients we observed here are more reflective of a region’s hierarchical position than simply its distance from neighboring regions. E.g., if two regions are anatomically distal from each other but close to each other in the processing hierarchy (e.g., hOc3d and hOc3v), a distance model would predict that they should have different expression magnitudes in either gradient. However, a hierarchy model would predict that they have similar expression magnitudes. Thus, if our observed gene gradients are truly related to a processing hierarchy, then our ability to predict one gene gradient’s expression magnitude from the other gradient should still be significant if inter-regional distance is included as a regressor (S3 Fig). We find that this is the case. Predicting the descending-gradient expression magnitude using the ascending gradient (91.4% variance explained) is only marginally improved with the additional regressor of distance (91.6% variance explained), and the coefficient of distance (coefficient = 0.01, *t* = 2, *p* = 0.043) is far smaller than that of expression magnitude (coefficient = 1.006, *t* = 32.14, *p* < 0.0001). Thus, macroanatomical proximity contributes a small amount to, and is not the driving factor of, the ordering of areas within the dendrogram. Finally, to evaluate the probability of reproducing the exact ordering of the transcriptomic visual hierarchy by chance, we used a bootstrap approach (*n* = 10,000; see Materials and Methods), which shuffles the gene expression profile within each cROI on each bootstrap. When implementing this bootstrapping approach, we find that the ability of the top genes to correctly order the cROIs is highly significant (*p* < 0.00001).

### Opposed genetic gradients correlate with cortical thickness and myelination

**Fig 4:**
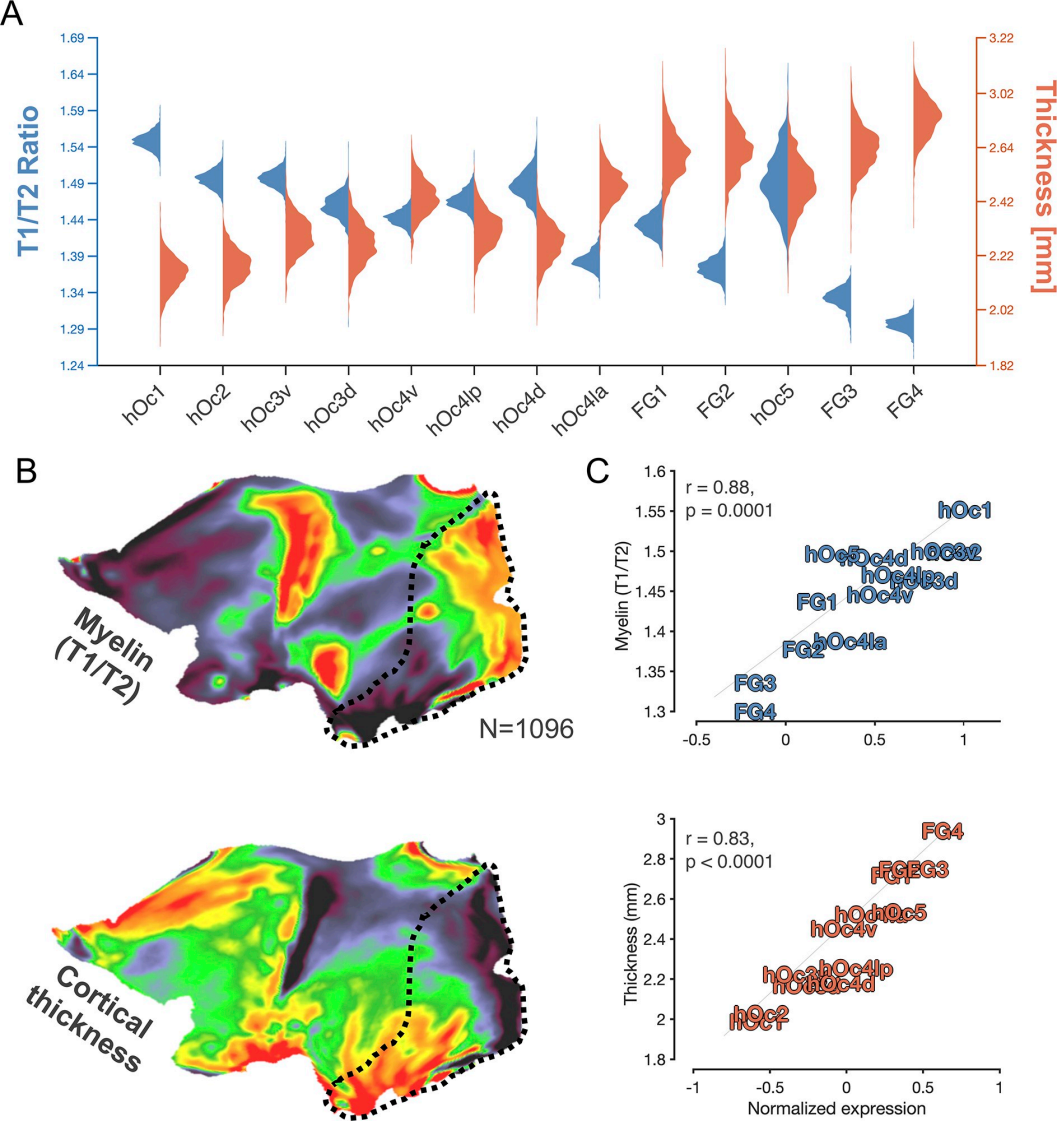
Ascending- and descending-gradient genes mirror inter-regional differences in cortical thickness and myelination. (A) Violin plots depicting the distribution of T1w/T2w ratio intensity, which is a metric reflective of tissue contrast enhancement associated with myelin content, in blue and cortical thickness in orange across HCP subjects (*n* = 1,096) in cROIs of the transcriptomic processing hierarchy. (B) Cortical flat maps depicting the average T1w/T2w ratio (top) or cortical thickness (bottom) across HCP subjects. Black-dotted outline highlights visual cortex. (C) Top: correlation plot between the average “myelin” content (T1/T2 ratio) in cROIs and average expression magnitude of genes included within the descending gradient. Bottom: same as above but for the average cortical thickness of a given cROI and expression magnitude of genes included within the ascending gradient. Thickness and myelination data can be downloaded from the HCP: http://www.humanconnectome.org/study/hcp-young-adult/data-releases. cROI, cytoarchitectonic region of interest; FG, fusiform gyrus; HCP, Human Connectome Project; hOc, human occipital; hOc d, hOc dorsal; hOc la, hOc lateral anterior; hOc lp, hOc lateral posterior; hOc v, hOc ventral; T1w, T1-weighted; T2w, T2-weighted.

To help elucidate the cortical mechanisms that are correlated with the two opposed gene gradients identified in the previous analyses, we next asked what anatomical features of the cortex covary with expression patterns. We specifically focus on two anatomical measures: myelination and cortical thickness. We focus on the former given previous findings demonstrating that V1 (hOc1) is a primary sensory region that is heavily myelinated at birth [49], while high-level visual areas demonstrate delayed and protracted myelination [31]. We focus on the latter given previous findings demonstrating that the thickness of the cortical sheet [50] distinguishes early sensory regions from regions located within higher-level association cortex. We used data from the Human Connectome Project (HCP) [51], which is a large database containing MRI scans from 1,096 adults and provides T1-weighted (T1w) and T2-weighted (T2w) scans that can be used to derive both measures of cortical thickness and a proxy of cortical myelin content (e.g., the ratio of T1w/T2w scans, which is a measure of tissue contrast enhancement related to myelin concentration; see Materials and Methods). We thus aligned individual subject maps of cortical thickness and maps of the ratio of T1w/T2w using CBA to the FreeSurfer average surface, to which the cROIs were also previously aligned using a similar procedure [26,31]. Then, in each cROI, we computed the average thickness or T1w/T2w ratio in each HCP subject. Illustrated in Fig 4A, we find that the T1w/T2w ratio produces a descending gradient as one ascends from hOc1 to FG4. Cortical thickness, in contrast, produces an ascending gradient from hOc1 to FG4. Distributions of cortical thickness and T1/T2 ratio are strikingly nearly Gaussian across subjects. An ANOVA with grouping variables of gradient type (T1w/T2w, thickness) and cROI reveals significant main effects of both variables (F-values_(1,12)_ > 50, *p*-values < 0.00001), as well as a significant gradient type × cROI interaction (F_(1,12)_ > 50, *p* < 0.00001). To visualize these anticorrelated gradients, we include cortical flat maps of average T1w/T2w ratio gradients and cortical thickness gradients from all 1,096 HCP subjects in Fig 4B.

Additionally, we directly examined the relationship between each cortical metric relative to each expression gradient, which resulted in two main findings. First, this approach revealed that a given cytoarchitectonic region’s T1w/T2w ratio is positively correlated with the expression level of descending-gradient genes (r(13) = 0.88, *p* = 0.0001; Fig 4C, top). Second, this approach also revealed that ascending-gradient gene expression levels are positively correlated with cortical thickness across cROIs (r(13) = 0.83, *p* < 0.0001; Fig 4C, bottom). Because cortical myelination is a factor that can influence in vivo estimates of cortical thickness from T1w images (because more myelin in deeper layers of the cortex near the gray–white boundary can “whiten” those voxels at the boundary, which can shift the estimated boundary of cortex further towards the pial surface), we produced a stepwise regression model including both T1w/T2w values and ascending-gradient expression levels as predictors of cortical thickness. When both terms are included, ascending-gradient gene expression still accounts for a significant portion of thickness variation across cROIs (coefficient = 0.55, *p* = 0.0004).

To gain further insight into the link between the ascending gradient and cortical thickness, as well as the descending gradient and myelination processes, we performed gene ontology (GO) enrichment analyses [52,53]. This approach revealed that, as a whole, the ascending-gradient gene cluster contains genes that primarily play a role in synaptic regulation and ion transport, implicating these genes in cell-to-cell signaling, which likely underlies their tight relationship to the thickness of cortical layers. Descending-gradient genes, in contrast, play a strong role in macromolecule metabolic processes. Myelin is one of the most ubiquitous macromolecules in the human brain, and its decreased presence in the cortex as one traverses further from hOc1 into ventral temporal cortex (VTC) is likely under the control of multiple genes within the descending-gradient cluster, given their strong correlation. To complement the GO analysis, we also performed a hypergeometric test to assess the extent to which changes in genetic transcription of these top 200 genes resulted from changes in the relative number of the underlying cellular populations between cytoarchitectonic regions. Using data from Lake and colleagues [54], which identified a list of 830 unique genes that were capable of distinguishing neuronal subtypes through single-nucleus RNA sequencing, we asked what proportion of our top 200 genes overlapped with these cell-type specific genes and whether this overlap was significantly above chance. We find that 31 of our 140 ascending-gradient genes and 12 of our 60 descending-gradient genes are genes whose expression magnitudes are capable of distinguishing neuronal subtypes as identified by Lake and colleagues (see S1 Table). A hypergeometric test confirms that this overlap is significantly (12.7 standard deviations) above chance. Thus, the expression differences between cROIs of 21.5% of our genes likely arises from inter-regional changes in neuronal subtypes as one traverses this transcriptomic hierarchy of visual areas.

### Gene expression gradients emerge at unique points in human development

**Fig 5:**
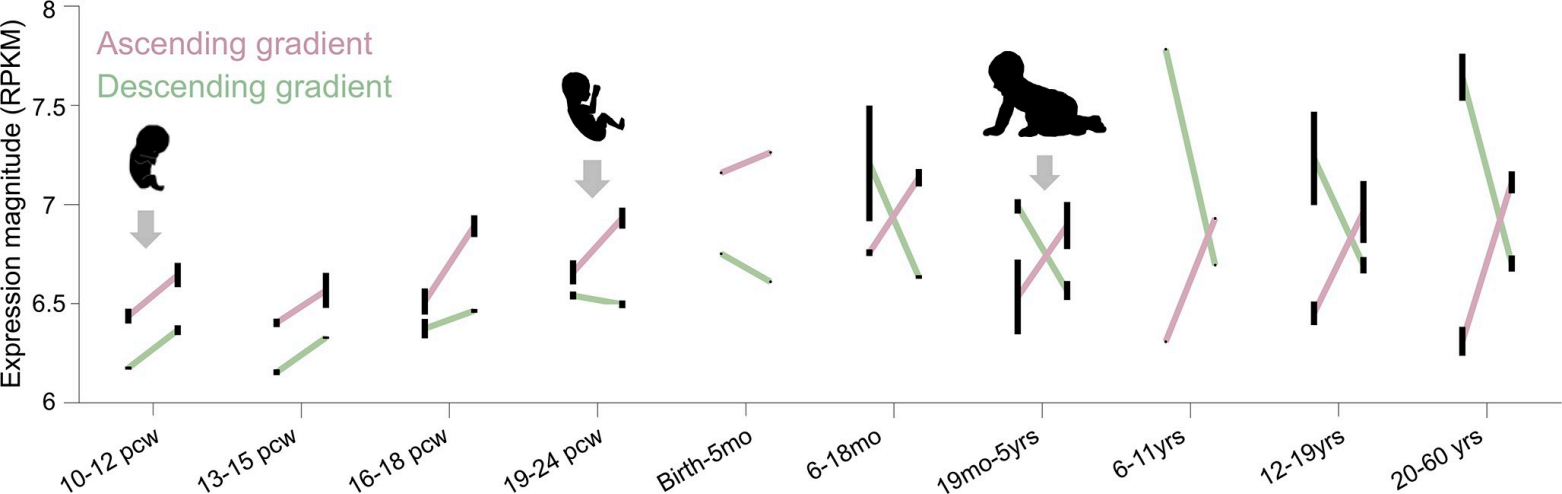
Ascending and descending gene gradients emerge at different points in human development. The two gene gradients derived from the top 200 differentially expressed genes in adults (ascending-gradient expression magnitudes shown in pink and descending-gradient magnitudes in green) show differences in their expression levels across development in early (pericalcarine cortex, left points in each line) and late (VLTC, right points in each line) visual cortex. See the following hyperlink for BrainSpan’s atlas detailing ROI definitions: http://www.brainspan.org/static/atlas. At the earliest time point (10 pcws), the ascending gradient (pink) shows an increasing expression profile from early to late visual cortex. However, the gradient genes that will show a descending expression profile (green) in adulthood also show an increasing profile at this stage of development. It is not until 19–24 pcws that the two gene gradients diverge in slope. Error bars reflect standard error across donor samples. Gestational transcription data can be downloaded from the Allen Institute’s BrainSpan project: www.brainspan.org. pcw, postconception week; ROI, region of interest, RPKM, reads per kilobase million; VLTC, Ventrolateral Temporal Cortex.

Given the facts that a) gradients are a fundamental principle underlying the developmental biology of many systems [55] and b) genes are dynamically expressed not only across tissue compartments but also throughout the life span, we asked at what point these gene gradients emerge during human development. To pinpoint the developmental trajectories of these gene expression gradients, we leveraged the developmental transcriptome of the BrainSpan [56] atlas of the developing human brain (http://brainspan.org), which characterizes the expression magnitude of the same gene targets as in our previous analyses. While the cortical locations sampled in this data set were sparser, the early visual (pericalcarine, which includes mostly hOc1) and late visual cortex (VTC, which includes the four FG areas; see Materials and Methods) were sampled across 42 brains ranging from prenatal to adulthood. Though this coverage is sparser than that of the previous analyses, which covered all 13 cROIs, it is a) sufficient enough to compare the early (e.g., hOc1) versus the late (e.g., the four FG areas) stages of the transcriptomic hierarchy and b) sensitive enough to detect a difference between these two stages because hOc1 and the four FG areas are the most differentiable based on their mean gene expression magnitudes (Fig 4). We hypothesized that opposing gene gradients (e.g., higher expression level in hOc1 compared to VTC and vice versa) should emerge prior to birth. To test this hypothesis, we first aimed to replicate our prior analyses by calculating the mean expression levels of the top 200 genes identified from the AHBA data in the 20–60 year old tissue samples from the BrainSpan atlas, which were closest to the age range used in the previous analyses in Figs 1–4. Illustrated on the far right of Fig 5, the ascending-gradient genes are expressed more in VTC compared to hOc1 (forming a positive slope), while descending-gradient genes are expressed most in hOc1 and least in VTC (forming a negative slope). Because this replicated our main finding from the analyses in Figs 1–4, we proceeded to examine the expression patterns for ascending- and descending-gradient genes at earlier developmental timepoints. We performed an ANOVA on the expression slopes (the difference between VTC and hOc1 expression magnitudes) with grouping variables of gene cluster and developmental time point, revealing a significant interaction (F(1,9) = 12.08, *p* < 0.0001). Surprisingly, we find that those genes constituting the ascending gradient demonstrate a positive slope at the earliest gestational time point (10–12 postconception weeks [pcws]) but that those genes belonging to the descending gradient have yet to reach their adult-like expression pattern and are instead expressed more highly in VTC than hOc1. It is not until 19–24 pcws that the descending-gradient slope diverges in sign from the ascending-gradient expression pattern. Furthermore, it is not until early childhood (before 5 years of age) that the two expression gradients achieve their adult-like mean expression values and characteristic crossover pattern.

### Only a subset of genes contribute to common ascending and descending transcriptomic gradients in humans and macaques

The emergence of different cortical structures during gestation is thought to mirror their different evolutionary time points [57]. Indeed, a key distinction of humans from other primate species is the expansion of the cortical sheet [58,59], whereby many structures extant in human high-level visual cortex, such as the FG and its constituent cytoarchitectonic regions, do not exist in the brains of NHPs. These structures emerge later in the womb than those that are evolutionarily preserved with NHPs—e.g., the calcarine and parieto-occipital sulci [60]. To see whether a) ascending and descending transcriptomic gradients exist in macaques and b) whether these transcriptomic gradients are linked to the same genes between species, we examined the expression pattern of the same ascending- and descending-gradient genes in the macaque monkey [61] using a transcriptome analysis performed similarly to the AHBA and BrainSpan data sets. Like the BrainSpan data, the NHP data set did not sample the cortex with the same density as the AHBA but did sample early visual cortex (V1 and V2) in addition to an area in high-level visual cortex (area TE [62], a high-level visual area in macaque inferotemporal cortex), which is considered to be homologous to human VTC and that we refer to as “late” visual cortex for the present analyses (Materials and Methods). We hypothesized that should the two opposed gene clusters be conserved in the adult macaque, then genes within a given gradient should show clear and consistent slopes (either positive for ascending-gradient genes or negative for descending-gradient genes) from early to late visual cortex.

**Fig 6:**
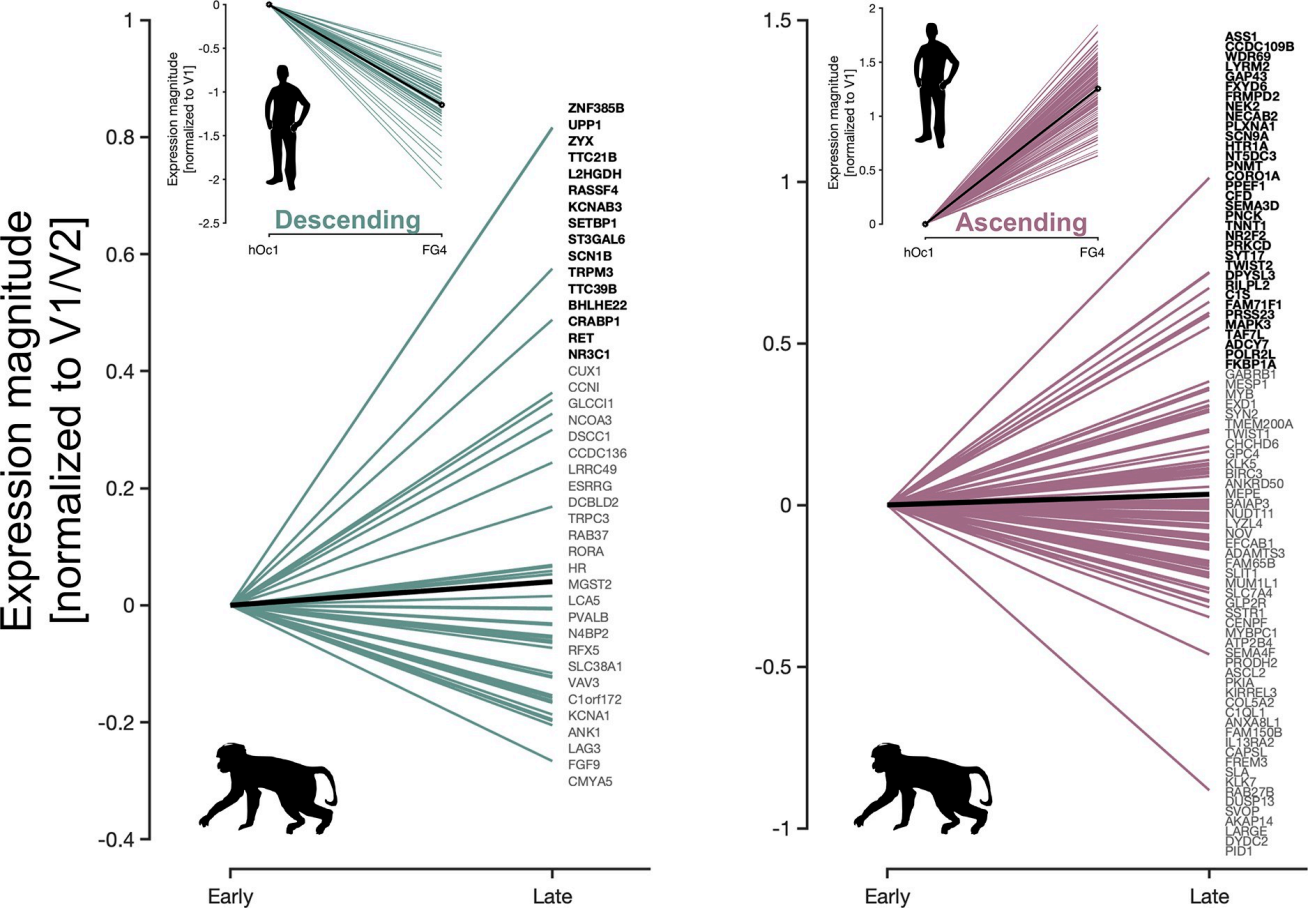
Homologous genes in macaques do not form ascending and descending gradients as in human visual cortex. Lines depicting the slope of transcription magnitude relative to early visual cortex in the macaque monkey for each gene. Green lines (left) are the expression slopes of homologous genes that were identified in humans as belonging to the descending gradient (see upper inset for expression slopes of the same genes in humans from hOc1 to FG4). Pink lines (right) are the expression slopes of genes that form the ascending gradient in humans. Because of sparser tissue sampling in the macaque compared to the human data set, early visual cortex was taken to be any tissue sample residing in either V1 or V2, while those in late visual cortex were taken from area TE, a region thought to be homologous to the FG cytoarchitectonic regions in humans. Solid black lines denote the mean slope. Individual gene names are listed on the right of each plot in descending order of slope (top corresponds to most positive line, bottom corresponds to most negative). Gene names bolded in black show positive slopes in expression magnitude from early to late processing stages. Light gray gene names show negative slopes. NHP transcriptional data can be downloaded here: http://www.blueprintnhpatlas.org/. FG, fusiform gyrus; hOc, human occipital; NHP, nonhuman primate; TE, a high-level visual area in macaque inferotemporal cortex.

Our analyses yielded three main findings. First, we find that mean expression magnitudes (averaged across all genes) are higher in late compared to early visual cortex for genes that form the ascending-gradient in humans, which indicates that the ascending gradient is also identifiable in adult macaques (Fig 6, right; black line depicts mean slope across all genes; *t* test comparing tissue samples in early versus late visual cortex: *t*(50) = 4.53, *p* < 0.0001). Second, we find that mean expression magnitudes are also higher in late compared to early visual cortex (Fig 6, right; black line; *t* test comparing tissue samples in early versus late visual cortex: *t*(50) = 4.35, *p* < 0.0001) for genes that form the descending gradient in humans, which indicates that the descending gradient is not identifiable in adult macaques—at least, using the genes belonging to the descending gradient in humans. Third, within each group of genes, a subset actually shows positive slopes in expression magnitude, and another subset shows negative slopes in expression magnitude, which does not occur in humans (insets in Fig 6). Thus, macaques also have a subset of genes that are differentially expressed between early and late visual processing stages, but these genes are distributed across genes from both ascending and descending gene gradients in humans. Thus, our analyses show that only a subset of genes contribute to common ascending and descending gradients across species.

## Discussion

Leveraging recent advances and shared data sets of human cytoarchitectonic areas in occipitotemporal cortex [7–13] and maps of gene expression across the cortical sheet [43,56,61], we demonstrate that the consistent anatomical layout and arealization of human visual cortex across individuals is correlated with two opposed gene expression gradients: one whose expression magnitude increases from posterior to anterior cortical areas and another whose expression magnitude decreases from posterior to anterior cortical areas. The former gradient correlates with the thickness of cortex, while the latter gradient correlates with the ratio of T1w/T2w values, thought to be related to cortical myelin content. These findings unveil a novel, to our knowledge, transcriptomic hierarchy of visual cortex and mirror recent work observing transcription gradients across the human [41] and mouse [48] cortical sheet. The present results build on the relationship between cortical hierarchies and transcriptomic gradients recently identified by Burt and colleagues [41] and show that two opposing genetic gradients contribute to the arealization of human visual cortex in a manner that is consistent with the proposed functional hierarchy of visual cortex (Table 1). Further, when examining the developmental trajectory of these transcription gradients, the ascending gradient emerges earlier in the gestational process during the first trimester compared to the descending gradient, which does not emerge until the second trimester. Finally, when examining the transcription of homologous genes in the macaque, we find that only a subset of genes contribute to common ascending and descending gradients across species. In the sections below, we 1) relate transcriptomic to functional hierarchies of human visual cortex, 2) compare transcriptomic and cytoarchitectonic hierarchies of human visual cortex, and 3) discuss the emergence of the transcriptomic hierarchy developmentally and between species.

**Table 1:** Relating cytoarchitectonic areas of the transcriptomic hierarchy to function. Left: Names of the cROIs from the Jülich Atlas. Middle: fROIs that are located within each cROI. fROI names in standard typeset have been empirically validated, while those fROIs that are italicized have been proposed to be located within a given cROI but have not yet been empirically validated. Right: The general type of visual processing typically associated with the fROIs and cROIs.

cROI	fROI	Type of Visual Processing Commonly Associated with each Region
hOc1	V1	low-level visual processing
hOc2	V2	low-level visual processing
hOc3v	V3v	low-level visual processing
hOc3d	V3d	low-level visual processing
hOc4v	hV4, VO-1	color, object form, surfaces
hOc4lp	*LO-1*	3D object form and surfaces
hOc4d	*V3AB*	depth perception, 3D object structure, motion perception
hOc4la	*LO-2*	object form
FG1	VO-1	object and color perception
FG2	pFus-faces (FFA-1), pOTS-words (VWFA-1)	face and word perception
hOc5	hMT+ (V5), TO-1, TO-2	motion perception
FG3	VO-2, PHC-1, PHC-2, CoS-places (PPA)	navigation of complex environments, perception of colors/objects
FG4	mFus-faces (FFA-2), mOTS-words (VWFA-2), OTS-bodies (FBA)	memory and perception of faces, bodies, words

**Abbreviations:** CoS, collateral sulcus; cROI, cytoarchitectonic region of interest; FBA, fusiform body area; FFA, fusiform face area; FG, fusiform gyrus; fROI, functional ROI; hMT+, human motion-selective complex; hOc, human occipital; hV4, the fourth visual area in human visual cortex; LO, lateral occipital; mFus, middle Fusiform; mOTS, middle occipitotemporal sulcus; pFus, posterior Fusiform; PHC, parahippocampal cortex; pOTS, posterior occipitotemporal sulcus; PPA, parahippocampal place area; TO, tempora-occipital; VO, ventral-occipital; VWFA, visual word form area; V3AB, the combination of two visual areas (V3A and V3B) in human visual cortex located anterior to the dorsal portion of the third visual area.

### Relating transcriptomic and functional hierarchies of human visual cortex

Using CBA and cross-validation procedures, recent studies have examined the correspondence between functional regions and cytoarchitectonic areas across the human visual hierarchy [26,63,64]. These studies found that in some cases, there is close to a one-to-one mapping between cytoarchitectonic areas and functional regions (e.g., hOc1 and V1, as well as hOc4v and the fourth visual area in human visual cortex [hV4]), while in other cases, there are many functional regions located within one cytoarchitectonic area (e.g., FG4 contains regions selective for three different categories: faces, bodies, and words). As illustrated in Table 1, we directly relate the position of each cytoarchitectonic area within the transcriptomic hierarchy to known functional regions that are located within each cytoarchitectonic area. Functional insights may be gleaned from the cytoarchitecture of each area, and in turn, the computations produced by the areas can be related to the position within the transcriptomic hierarchy. E.g., hOc1, which contains functionally defined V1, is positioned furthest from FG3 and FG4, which each contain regions selective for different categories such as places and faces, respectively. In turn, the position of hOc1 relative to FG3 and FG4 within the transcriptomic hierarchy is consistent with the earliest and latest stages of the functional hierarchy of visual cortex because V1 is considered the earliest stage and category-selective regions are considered one of the latest. Likewise, unexpected insights also occur when relating cytoarchitectonic and functional regions within specific subclusters of the transcriptomic hierarchy. E.g., FG2, which contains regions selective for faces and words, is proximal to FG3 and FG4 in the transcriptomic hierarchy but in a separate subcluster with hOc5, which contains a motion-selective region (human motion-selective complex [hMT+]). The fact that face- and motion-selective regions are within the same subcluster is unexpected, considering category-selective regions are often considered to be within later processing stages after motion-selective regions such as hMT+ and V3A [1,65]. Nevertheless, it is not surprising when examining basic visual properties of these regions, which we unpack further in the last paragraph of this section.

The reader can glean additional insights by relating the position of the cytoarchitectonic area in the transcriptomic hierarchy and the functional regions located within each area (**Table 1**). E.g., even though regions selective for the same category such as faces (posterior Fusiform [pFus]-faces and middle Fusiform [mFus]-faces, located in FG2 and FG4, respectively) are located on the same macroanatomical structure (the FG), they are transcriptomically differentiable. Thus, while the present findings indicate that differences in anatomical features such as thickness and myelination likely contribute to the positioning within the transcriptomic hierarchy, functional features of the cytoarchitectonic areas likely also contribute.

**Fig 7:**
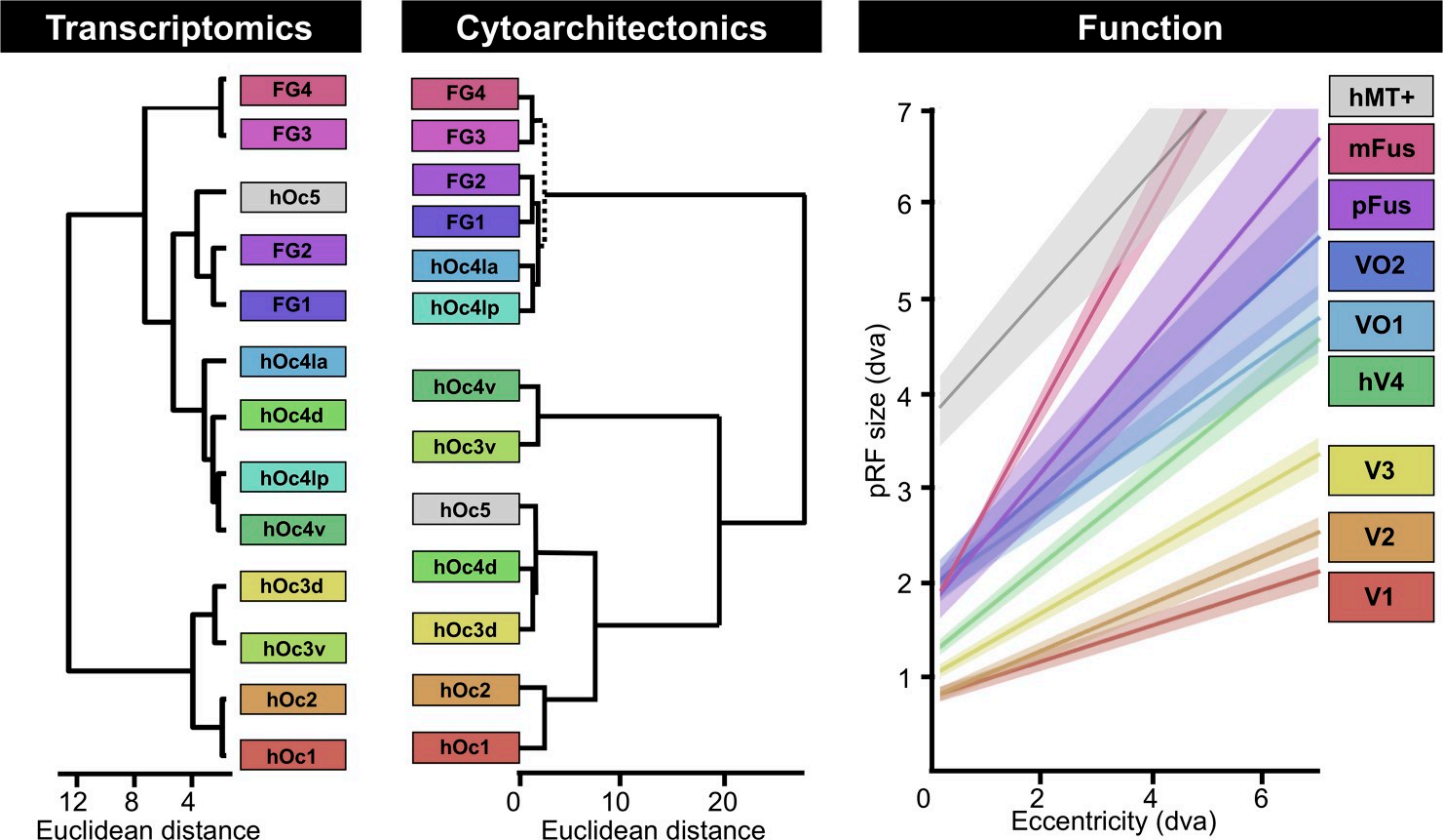
Comparison among hierarchies of areas in human visual cortex generated by transcriptomics, cytoarchitectonics, or functional properties. Left: connective models of visual regions composing processing hierarchies as measured with either transcription (data from Fig 3B) or cytoarchitecture (a combination of dendrograms produced by Malikovic and colleagues [11] and Lorenz and colleagues [13]). Color along the rainbow spectrum corresponds to the region’s numerical rank according to name (red, orange, yellow, green: hOc1, hOc2, hOc3, hOc4, etc.). hOc5 was not colored because of the fact that only a small number of samples (*n* = 2) were located within this area. Dotted line denotes where branch from Lorenz and colleagues [13] was added to the dendrogram from Malikovic and colleagues [11]. Right: Linear fits relating pRF size and eccentricity from voxels located in visual field maps (V1 through VO-2), face-selective regions (pFus-faces and mFus-faces), as well as a motion-selective region (hMT+) of the visual hierarchy. Regions are colored according to the cytoarchitectonic region in which they reside based off of previously published work (Table 1). For all regions except mFus-faces, solid lines denote mean line of best fit across 40 subjects from Gomez and colleagues [35, 72] (shaded region is standard error across subjects). For mFus-faces, the solid line and shaded region are the mean and 68% confidence intervals across the three subjects from Kay and colleagues [70]. The slope relating a pRF’s eccentricity to its size increases as one ascends the visual hierarchy. Note that the relationship between pRF size and eccentricity in hMT+ is more similar to pFus-faces and mFus-faces than other visual regions tested in these previously published studies. Because hMT+ is located within hOc5 and pFus-faces is located within FG2, the adjacent positioning of these two regions within the same subcluster in the transcriptomic hierarchy may be related to similar pRF properties between these two regions (see Discussion). FG, fusiform gyrus; hMT+, human motion-selective complex; hOc, human occipital; hOc d, hOc dorsal; hOc la, hOc lateral anterior; hOc lp, hOc lateral posterior; hOc v, hOc ventral; mFus, middle Fusiform; pFus, posterior Fusiform; pRF, population receptive field; VO, ventral-occipital.

In addition to these descriptive functional insights, recent evidence also enables quantitative insights relating transcriptomic and functional hierarchies of visual cortex. Specifically, a well-known computational unit of visual cortex is the receptive field, which is the region of the visual field within which a visual stimulus elicits a response from a neuron [66]. fMRI enables researchers to measure population receptive fields (pRFs), which are the regions of visual space that stimulate a voxel [67,68]. A textbook property of the visual system is that RFs, and in turn pRFs, start out small in V1 (hOc1) and get larger with each stage of the hierarchy, and furthermore, that this increase at each stage of the hierarchy also scales with eccentricity [68]. Additionally, previous work demonstrates that visual field map borders often serve as cytoarchitectonic borders, especially differentiating the first and second visual areas (see Amunts and Zilles [69] for a review). In Fig 7, we provide pRF measurements from regions that were recently published [70,71] and that are located in different positions of the transcriptomic hierarchy. As is likely obvious to the reader, these functional regions are not only located within different cytoarchitectonic areas that have different positions in the transcriptomic hierarchy (**Table 1**), but they also have different pRF-eccentricity relationships, which is considered a quantitative feature at the foundation of the visual processing hierarchy. Additional insights are further gleaned regarding the transcriptional positioning of hOc5 in a subcluster with FG2 and FG1. Specifically, the relationship between pRF and eccentricity in hMT+ [72] (located in hOc5) is more similar to pFus-faces (located in FG2) and mFus-faces (located in FG4) than the other visual regions that were measured in these previously published studies [35,70] (Fig 7). Thus, while it may seem counterintuitive that a cytoarchitectonic region involved in motion processing is transcriptomically similar to cytoarchitectonic areas involved in face and object processing (FG2 and FG1), this similarity may be based on the similarity of pRF size within these regions. Taken together, we propose that the position in the transcriptomic hierarchy identified here is linked to the position in the functional hierarchy of visual cortex and that a subset of genes from either the ascending or descending gradient (or both) are related to pRF size. We also propose that additional functional gradients that exist across the human visual processing hierarchy likely emerge and develop from structural gradients endowed by the genetic gradients identified in the present study.

### Linking transcriptomic and cytoarchitectonic hierarchies of human visual cortex

Though we examined transcriptomic samples from different cytoarchitectonic areas, the analyses and interpretation of our results indicate that cytoarchitectonic differences among areas are not the only features contributing to the positioning of areas in the transcriptomic hierarchy. E.g., functional features (Table 1; Fig 7), myelin content (Fig 4), and cortical thickness (or thinness; Fig 4) also contribute to the positioning of areas within the transcriptomic hierarchy. Nevertheless, because previous studies performed hierarchical analyses of the cytoarchitectonic properties of the areas used in the present study, a useful exercise is to directly compare the resulting hierarchies produced from the two approaches. As illustrated in Fig 7, both approaches generally cluster early, middle, and late processing stages from one another. However, there are interesting differences in the positioning of areas within each hierarchy. E.g., the two clusters produced by the transcriptomic dendrogram at the highest level separate hOc1, hOc2, hOc3d, and hOc3v from the four areas within the hOc4 cluster, hOc5, and the four FG areas. In contrast, the two highest clusters produced by the cytoarchitectonic dendrogram separate the four FG areas along with hOc4la and hOc4lp from all other areas. The reader can also appreciate the differences in the subordinate clusters as well. Despite these differences, each area is positioned in a similar place within the transcriptomic and cytoarchitectonic hierarchies of visual cortex. E.g., hOc4lp is positioned as the fifth or seventh area, while FG2 is positioned as the 10th or 11th area, from hOc1 in the transcriptomic and cytoarchitectonic hierarchies, respectively. The area that is situated most differently between the two hierarchies is hOc5. We emphasize that there are very few transcriptomic samples in hOc5 (S1 Fig), and thus, we are limited in interpreting the meaning behind this difference. Instead, the main conclusion from qualitatively comparing these hierarchies to one another is that there is not a one-to-one correspondence between the two, which supports the conclusion that strict cytoarchitectonic differences among areas are not driving the clustering of the transcriptomic hierarchy of human visual cortex. Instead, the present results indicate that multiple anatomical and functional features are contributing to (or likely result from) the transcriptomic hierarchy of human visual cortex.

Despite these conclusions, it is also interesting to hypothesize whether some cytoarchitectonic features are contributing to the transcriptomic hierarchy more than others. E.g., cell density across cortical layers is a major factor contributing to the assessment of cytoarchitecture and in some cases results directly from expression levels of specific genes like paired box 6 (PAX6) [73] during development. It is well known that cell density is highest in visual areas compared to the rest of cortex. Additionally, it is also known that cell density is highest in V1 [74] and decreases across the human visual hierarchy [75], with cell density in regions of the FG being about one-third of that of V1 (see [76]). While decreasing cell body density could in theory explain some of the variation in the decreasing expression trend of the descending-gradient genes, changes in the relative number of specific cell subtypes within a given cortical region could explain inter-regional transcription differences. Indeed, 21.5% of the genes we identified here were also identified by Lake and colleagues [54] as genes that distinguish 8 separate excitatory and 8 separate inhibitory neurons in the human cortex, suggesting that some of the hierarchy of transcription we observe here could be linked to changes in the underlying neuronal population as one traverses the visual hierarchy. E.g., previous work [69,77,78] shows that gamma amino-butyric acid (GABAb) receptor density can increase from hOc1 to hOc4v despite decreases in cell density from hOc1 to hOc4v. Altogether, our present results indicate that a) multiple anatomical and functional features across spatial scales are contributing to the construction of the transcriptomic hierarchy of human visual cortex and b) differences in cell density among cytoarchitectonic areas likely contribute to, but likely do not drive, the construction of the transcriptomic hierarchy of the human visual system.

### The emergence of the transcriptomic hierarchy of visual cortex developmentally and between species

The consistent relationship among cortical folding, cytoarchitectonic transitions, and functional regions at different stages of the visual hierarchy in human adults is likely related to the fact that the two genetically opposed expression gradients identified in the present study are shared across individuals. Interestingly, these transcriptomic gradients emerge at different points in human development. While we acknowledge that our present developmental and between-species analyses are limited by small sample sizes from shared data sets, the developmental and evolutionary insights from these analyses generate powerful hypotheses for future studies utilizing larger sample sizes.

In terms of development, we propose that there is an early and a late developmental phase underlying the transcriptomic hierarchy of visual cortex. The early phase emerges in the first trimester, during which microanatomical differences that make up the unique cytoarchitecture in each stage of the visual processing hierarchy are likely controlled primarily by the ascending-gradient genes. The late phase emerges in the second trimester, linked to descending-gradient genes, during which microanatomical differences that are both functionally relevant and contribute to the composition of the transcriptomic hierarchy of visual cortex are likely further refined. This is especially true within those regions located in macroanatomical structures that are hominoid-specific such as the FG. While our present evidence indicates that both gradients of the transcriptomic hierarchy emerge at different timepoints in gestation, we also propose that both gradients change postnatally, likely until children are well into their school-age years. This continued development would suggest that while genes aid in establishing areal differences in the visual hierarchy before birth, gene expression may also interact with visual experience and contribute to changes in the visual hierarchy well after birth, which can be examined in future research. The findings at hand also establish important groundwork for future research seeking to link mutations in genes belonging to a given gradient to specific regions of visual cortex. Indeed, as ongoing research links polymorphisms in specific genetic loci with structural variability in specific regions of cortex [79,80], individual variability in perceptual skills (i.e., reading ability, face recognition, etc.) or traits in certain disorders may be a promising avenue to begin linking genetic variability with experiential and cortical differences.

Between species, we observed that macaques also have a subset of genes that are differentially expressed between early and late visual processing stages. However, while some of the gradients formed by a given macaque gene could be predicted by its gradient of expression in humans, others could not. Thus, our analyses show that only a subset of genes contribute to common ascending and descending gradients across species. Concomitantly, the results from the present study indicate that while the hierarchy of visual areas in the macaque brain is often used as a model for the hierarchy of visual areas in the human brain [41,81–85], known differences between species in the localization, presence, and organization [86–92] of areas composing the visual hierarchy may be partly explained by transcriptomic differences contributing to dissimilar gradients across species. How transcription contributes to the functional and anatomical organization of the visual hierarchy, in addition to other functional hierarchies, in cortical locations that are present in humans and not macaques is a major topic for future research because it will likely provide key insights into human-specific aspects of high-level cognition.

### Conclusion

Combining cytoarchitectonic definitions of human occipitotemporal cortex with genetic transcription analyses of the cortex, we identified a transcriptomic hierarchy of human visual cortex that is explained by two opposing gene expression gradients: one that contains a series of genes with expression magnitudes that ascend from posterior (e.g., areas hOc1, hOc2, hOc3, etc.) to anterior cytoarchitectonic areas (e.g., areas FG1–FG4) and another that contains a separate series of genes that show a descending gradient from posterior to anterior areas. Not only do these findings help establish genetic correlates of areal differentiation and the consistency of brain organization across individuals, but they also identify a sparse subset of genes that form opposing expression gradients across the human visual processing hierarchy—gradients that a) seem to emerge at different points in human development and b) are different between humans and NHPs. These findings provide essential groundwork for understanding both the origins of cortical processing networks and the factors that can contribute to their maldevelopment. Future studies can focus on the genes identified in the present study to more closely examine their causal role during cortical development and potentially identify polymorphisms or mutations in these genes in known developmental deficits such as autism, dyslexia, and prosopagnosia.

## Materials and methods

### Data

All data analyzed in the present manuscript were curated from 5 freely available data sets that were acquired, shared, and approved according to the Ethics Committees of each institution:

1. JüBrain: http://www.fz-juelich.de/JüBrain/EN/_node.html;
2. AHBA: http://brain-map.org/;
3. HCP: http://www.humanconnectome.org/study/hcp-young-adult/data-releases;
4. BrainSpan Atlas: www.brainspan.org;
5. NIH Blueprint NHP Atlas website: http://www.blueprintnhpatlas.org/.

### JüBrain cytoarchitectonic atlas and ROI definition

The JüBrain cytoarchitectonic atlas consists of a set of cytoarchitectonic brain areas that are defined by an observer-independent analysis of laminar cell-density profiles in 10 human postmortem brains (https://www.fz-juelich.de/inm/inm-1/EN/Forschung/JuBrain/_node.html). Each area is represented by a 3D map that describes the maximum probability with which a certain cytoarchitectonic area can be assigned to a certain macroanatomical location in the brain [17, 69]. Presently, 100 areas have been mapped in the atlas, which covers about 70% of the brain. Among them, 13 visual areas located in occipitotemporal cortex have been delineated, including hOc1, hOc2, hOc3d, hOc3v, hOc4d, hOc4v, hOc4lp, hOc4la, hOc5, FG1, FG2, FG3, and FG4 (Fig 1). These 13 cROIs are the focus of the present study. Because the JüBrain cytoarchitectonic atlas has been aligned to the MNI305 (Colin 27) space and the AHBA has been aligned to the MNI152 space, the JüBrain cytoarchitectonic atlas was first linearly transformed into the MNI152 space using FMRIB's Linear Image Registration Tool (FLIRT) to align the cROIs to the AHBA.

### Human transcriptome data and analysis

#### AHBA

The gene expression data used in the present study were obtained from the AHBA, which is a publicly available atlas of gene expression and anatomy. The AHBA employs DNA microarray analyses to map gene expression from tissue samples taken broadly across human cortex. The data set is based upon the conglomeration of measurements from 6 postmortem human brains, although not all brains were sampled identically. In the end, cortical samples were acquired using macrodissection from every brain and submitted to normalization analyses to make measurements within and between brains comparable. Each sample was associated with a 3D coordinate from its donor’s MRI brain volume and its corresponding coordinate (*x*, *y*, *z*) in the MNI152 space. Each tissue sample was analyzed for the expression magnitude of 29,131 genes, with 93% of known genes being queried by at least 2 probes. For more details regarding how the microarray data were normalized, please see the following documentation: http://help.brain-map.org/display/humanbrain/documentation/.

#### Gene expression preprocessing

The collection and quality control of the gene expression data has been described previously (see online documentation link above). Here, we discuss the preprocessing of data for the study at hand. The raw microarray expression data for each of the six donor brains included the expression level of 29,131 genes profiled via 58,692 microarray probes. We implemented five preprocessing steps. First, probes were excluded that did not have either a) a gene symbol or b) an Entrez ID. This resulted in 20,737 genes. Second, the expression profiles of all the probes targeting the same gene were averaged. Third, in order to remove variability in gene expression across donors, gene expression values were normalized by calculating z-scores separately for each donor. Fourth, and as described above, samples were assigned to cROIs based on MNI152 coordinates. Specifically, samples were assigned to cROIs based on the Euclidean distance between the sample and a cROI. Fifth, because previous studies did not identify significant interhemispheric transcriptional differences, data from both hemispheres were combined. As a result, a total of 331 samples were mapped to the 13 cROIs in occipitotemporal cortex across all donors. The number of samples varied across cROIs (see S1 Fig).

#### Gene selection

In order to identify the genes that were expressed differentially across areas within human visual cortex, we first divided the 13 cROIs into groups with equal samples (S1 Fig). This was done in order to equalize (as closely as possible) the number of samples in a given group for the purposes of statistical analysis. We divided the samples from the 13 cROIs into 4 groups: hOc1, [hOc2 and hOc3], [hOc4d, hOc4v, hOc4lp, hOc4la], and [FG1, FG2, FG3, and FG4]. hOc5 was excluded because only 2 samples were included within that region of cortex. Once the cROIs were divided into groups, for each gene, we ran a 1-way ANOVA with cROI group as a factor. We ranked the genes according to the *p*-values from the ANOVA and selected the top approximately 1% (200) of genes for further analysis. This thresholding approach was taken in order to avoid including genes that may result in significance simply as a function of multiple comparison. The names of these 200 genes are listed in S1 Table and colored according to gradient membership (e.g., ascending or descending).

To ensure that our results were not limited to the method used to select the genes, we repeated our gene selection analyses using a PCA on the full gene expression data from the 13 cROIs. As illustrated in S2 Fig, our results are not dependent on the method used to select the genes. With either method, two opposing gradients are identified. With the PCA approach, the two gradients represent positive and negative directions on the first PC (PC1 in S2 Fig), which explains 33.8% of the variance. The PCA is described in detail in S2 Fig.

#### Exploring the relationship of gene expression among cROIs

In order to further explore the pattern of gene expression across the cROIs in human occipitotemporal cortex, the microarray data were first averaged across all samples from all donors within a cROI across both hemispheres. Accordingly, a 13 × 200 data matrix was generated (Fig 2B), which represents the expression pattern for the top 200 genes across the 13 cROIs. To evaluate the potential significance of the two visible gradients in this matrix (Fig 2B), we used a bootstrapping approach to evaluate its significance against an equally sized hypothesis matrix (Fig 2B) in which the expression gradient of each gene either linearly increased or decreased across cROIs according to gene gradient membership. To do so, we produced a null distribution through bootstrapping (*n* = 1,000) in which on each iteration, the cROI columns were shuffled within the observation matrix before correlating with the hypothesis matrix. This produced the orange histogram in Fig 2B. The correlation between the original (e.g., intact) observation matrix and the hypothesis matrix is demarcated with a red line in relation to this null distribution, where one can see it is significantly outside the null distribution. We also repeated this bootstrapping approach; this time, instead of shuffling, we only used a subset (e.g., 70%) of the observation and hypothesis matrices. This blue histogram is well outside the null distribution. Lastly, repeating the correlation between the two matrices by explicitly excluding hOc1/hOc2 and FG4 (the extrema of the gradients) still produces a correlation (dotted red line) that is significantly outside the null distribution.

We then used the observed matrix as the input to an agglomerative hierarchical analysis using Euclidean distances and the weighted-pair group method with arithmetic mean (WPGMA) in order to examine the relationship of gene expression among the cROIs in occipitotemporal cortex. Prior to clustering, the 13 × 200 matrix was sorted according to each cROI’s Euclidean distance from the expression pattern of hOc1. In this way, the ordering of cROIs along the x-axis in the resulting dendrogram is meaningful because it represents the distance from hOc1 at the level of dendrogram leaves. As a result, this dendrogram has rooted leaves, and the clusters at this lowest level are not rotatable as in other dendrograms.

To evaluate the significance of this dendrogram ordering, we evaluated how often such an ordering of these region can occur by chance. To accomplish this, we utilized a bootstrap approach with 10,000 iterations. On each bootstrap, within each cROI, we randomly shuffled the expression magnitudes of the 200 genes before submitting the cROI expression profiles to the agglomerative clustering algorithm. We then took the resulting ordering vector from the clustering algorithm (e.g., [2 11 5 3…7]) and calculated its Euclidean distance from the true ordering vector (e.g., [1 2 3 4 5…13]). We obtained the *p*-value as the resulting probability of observing a zero Euclidean distance from the resulting 10,000 samples.

Lastly, to quantify what role inter-regional distance played in the gradient expression of genes in relation to hierarchical ordering, we performed an analysis in which we assumed that if inter-regional distance was the major factor in explaining transcription variation from one region to the next (rather than hierarchical position), then predicting one gradient’s expression levels across cROIs using the other gradient’s expression levels, while regressing out inter-regional distance, should make the correlation diminish drastically. To this effect, we produced two matrices (13 cROIs × 13 cROIs). Illustrated in S3 Fig, one matrix contained values of inter-regional distances (measured as Euclidean distance from the central MNI coordinates of each cROI) and the other contained difference values in transcription expression of descending-gradient genes. We then performed stepwise regression to evaluate the relative contribution that hierarchy level (assessed using ascending-gradient gene expression levels) and inter-regional difference make in explaining gene expression across the visual hierarchy. We find that a given region’s genetic expression is much more strongly predicted by its hierarchical position rather than its anatomical distance from other regions of cortex (for further specifics of these analyses, please refer to the Results section “Clustering expression levels of the ascending and descending gradients produces an ordering of cytoarchitectonic regions reflective of a novel transcriptomic hierarchy in human visual cortex”).

### HCP measures of T1, T2, and cortical thickness

#### Data

Cortical maps of T1w intensity, T2w intensity, and cortical thickness were obtained from 1,096 participants included in the HCP [93]. In each subject, T1w and T2w scans were collected using 3-Tesla MRI. While not a direct measure of cortical myelination, the ratio of T1w to T2w scans removes MR-related image inhomogeneities to a given voxelwise signal that is thought to be sensitive to the influence of myelin and iron (which are strongly colocalized in cortex). Measures of cortical thickness were produced using the automatic tissue segmentation algorithm in FreeSurfer [50], version 6.0.0.

#### ROIs

Because T1w and T2w measurements, as well as measures of cortical thickness, were projected to individual HCP cortical surfaces, we transformed cROIs into each individual HCP subject using CBA by first transforming the cROIs to the FreeSurfer average surface and then transforming the cROIs into individual HCP surfaces to ensure that individual differences in cortical folding were respected in each subject. This methodology assures that the coupling between cytoarchitecture and cortical folding observed in the postmortem brains is maintained in living subjects [26,31].

#### Analyses

After aligning cROIs to each individual HCP participant, we then extracted the mean T1w/T2w value and mean cortical thickness value within each cROI for all 1,096 HCP subjects. Distributions of these means across subjects for all cROIs is illustrated in the violin plots of Fig 4A. We statistically examined the relationship between anatomical gradient (T1w/T2w, thickness) and cROI in two ways: 1) with an ANOVA (Fig 4A) and 2) the correlation between anatomical gradient and mean gene expression value (Fig 4C).

### Developmental transcriptome data and analyses

#### Data

The BrainSpan Atlas contains gene expression data taken from cortical structures spanning 13 stages of human development across 8–16 brain structures. The atlas queried the expression of 17,604 genes via DNA microarray analyses similar to the AHBA described above. We focused our analyses on transcriptome data from two ROIs: primary visual cortex and VTC. The primary visual cortex for intrauterine cases included samples taken in the vicinity of what would become pericalcarine cortex, including mostly V1. The VTC was taken from the region of cortex labeled as “Ventrolateral Temporal Cortex” (VLTC, http://www.brainspan.org/static/atlas), which would become inferior temporal cortex, which corresponds roughly to the four cytoarchitectonic ROIs labeled FG1–4 in adult cortex (Fig 1). Because these areas cannot be recognized before 10 pcws, the developmental data from the first two time points (1 and 2A) were excluded from further analyses. The gradient genes we identified from the AHBA data set (e.g., top 200) were then targeted for our analyses. See S2 Table for the stage division and the corresponding number of samples from this data set. Information about data preprocessing and normalization is available from the BrainSpan Atlas website (http://help.brain-map.org//display/devhumanbrain/Documentation).

#### Analyses

Using these data, we first validated whether the observed spatial gradients (ascending and descending) that we identified in the AHBA data set could be replicated from the adult time point in the BrainSpan data set. Once we verified that the ascending and descending-gradient genes demonstrated their expected expression levels in V1 and VTC, we then measured their expression magnitudes in the same ROIs in early time points (Fig 5). At each time point, we averaged gene expression (reads per kilobase million [RPKM]) across all samples and donors within a given ROI to obtain an average ascending or descending-gradient expression magnitude by time point.

### NHP transcriptome data and analyses

#### Data

The DNA microarray data for NHPs were from the “Transcriptional Architecture of the Primate Neocortex” study [61], a subproject of the NHP atlas. Samples were collected from 10 cortical areas using laser microdissection in four adult rhesus monkeys. Each area was assayed using Affymetrix GeneChip Rhesus Macaque Genome Arrays (19,050 genes, 52,865 probes; Thermo Fisher Scientific, Waltham, MA, USA). For the present analyses, transcriptome data from three ROIs were considered: V1, V2, and TE. For the sake of our analyses, V1 and V2 were grouped into a single ROI, which we refer to as early visual cortex in Fig 6. We also refer to data from TE as late visual cortex in Fig 6. The expression profiles of all the probes representing one gene were averaged within an ROI and across monkeys. As the cortex was sectioned via laser microdissection, sections from individual cortical layers were submitted to microarray analysis separately. In order to maximize the homology of analyses between humans and macaques, we averaged the data across all six layers in macaques. More complete descriptions of the experimental and data processing methods are available in protocol documents at the NIH Blueprint NHP Atlas website: (http://www.blueprintnhpatlas.org/).

#### Analyses

We first took the top 200 genes identified in adult humans and identified their NHP homologues using a gene-symbol–mapping table provided by the NHP atlas. 153 human genes had a match in the macaque gene set; the other 47 were likely not measured. 36 of these missing genes belonged to the ascending gradient (the larger of the two gradients), and 11 belonged to the descending gradient. The expression magnitude (RPKM) of all the probes representing a single gene were averaged together. Tissue samples within V1 and V2 were averaged together into an early visual cortex group. Tissue samples within area TE, a high-level visual cortical region homologous with human FG regions, were averaged together into a late visual cortex group. To better visualize the expression slope of an individual gene’s expression magnitude between early and late visual cortex, we normalized each gene’s expression magnitude in early and late visual cortex by subtracting its magnitude in early visual cortex from both values. We then plotted lines for each individual gene in Fig 6 whose slope denotes whether or not it ascends or descends in its expression magnitude from early to late visual cortex.

## Supporting information

S1 Fig Grouping tissue samples into cytoarchitectonic neighborhoods. Left: once the cROIs were aligned to the transcriptomic data (Materials and Methods), we quantified the number of tissue samples from the 6 postmortem brains included in the AHBA that were located within each cROI. The colors for each cROI are the same as in Fig 1B. Right: prior to gene selection, we first grouped expression samples into four cytoarchitectonic neighborhoods (differently shaded gray bars) because there were an unequal number of tissue samples in each cROI. The hOc4 grouping includes samples from hOc4d, hOc4lp, hOc4la, and hOc4v. The FG grouping includes regions FG1 through FG4. Given the small number of tissue samples from hOc5 (*n* = 2), we excluded it in the final ANOVA to avoid bias. Importantly, the identification of the ascending and descending gene gradients is not dependent on the method used for gene selection (S2 Fig). AHBA, Allen Human Brain Atlas; ANOVA, analysis of variance; cROI, cytoarchitectonic region of interest; FG, fusiform gyrus; hOc, human occipital.
(TIFF)

S2 Fig PCA of averaged transcription profiles captures the ascending and descending-gradient pattern in the first component. (A) We submitted the average expression magnitude of all genes within each of the 13 cROIs to PCA, the first component of which demonstrates a gradient of weighting scores, with posterior visual areas (e.g., hOc1, hOc2, etc.) mapping negatively onto this PC and anterior (e.g., FG2, FG3, etc.) visual regions mapping positively. (B) Histogram of Pearson correlation significance values (negative log-transformed) between each individual gene’s expression magnitude across the 13 cROIs with the scores of the first PC. Those genes with–log *p*-values exceeding 3 are highlighted in darker pink. (C) Linear fits summarizing the expression magnitude across cROIs of genes demonstrating either positive (warm colors, top) or negative (cool colors, bottom) correlations with PC1 scores. We took a stepwise approach, first including all genes whose–log *p*-values exceeded 3 in the average and then incrementally increasing the threshold until we only included genes in each group whose *p*-values exceeded 12, which included approximately 200 genes, equivalent to the group of 200 genes we chose in Fig 1. The magnitude of the positive or negative slope describing the expression gradients across cROIs increases as one includes more significantly differentially expressed genes. These analyses reveal that our results are not dependent on the method used to select the genes. With either method, two opposing gradients are identified. With the PCA approach, the two gradients represent positive and negative directions on the first PC, which explains 33.8% of the variance. cROI, cytoarchitectonic region of interest; FG, fusiform gyrus; hOc, human occipital; PCA, principal component analysis.
(TIFF)

S3 Fig Hierarchical position, but not anatomical position, is more predictive of genetic expression patterns. Left: inter-regional distances measured in MNI space between all cROIs of interest. Middle: inter-regional differences in the mean expression magnitude of descending-gradient genes. The descending-gradient matrix is the matrix whose values are being predicted by a stepwise regression, including the Euclidean distance and expression magnitude of ascending-gradient genes (right) as regressors. The beta-weights and significance of each regression are written beneath each matrix. cROI, cytoarchitectonic region of interest; MNI, Montreal Neurological Institute.
(TIFF)

S1 Table Gene symbols of the top 200 genes. Genes written in green belong to the descending gradient cluster, while those in pink belong to the ascending gradient cluster. Bolded gene names are those that were identified by Lake and colleagues [54] as differentiating specific neuronal subtypes.
(TIFF)

S2 Table The definitions of developmental stages and the corresponding number of samples in each stage. This information and relevant data can be found at brainspan.org. pcw, postconception week.
(TIFF)

## Abbreviations

AHBA: Allen Human Brain Atlas
ANOVA: analysis of variance
CBA: cortex-based alignment
CoS: collateral sulcus
cROI: cytoarchitectonic region of interest
FBA: fusiform body area
FG: fusiform gyrus
FLIRT: FMRIB Linear Image Registration Tool
fMRI: functional MRI
fROI: functional region of interest
GABAb: gamma-aminobutyric acid receptor
GLI: gray-level index
GO: gene ontology
HCP: Human Connectome Project
hMT+: human motion-selective complex
hOc: human occipital
hOc d: hOc dorsal
hOc la: hOc lateral anterior
hOc lp: hOc lateral posterior
hOc v: hOc ventral
LO: lateral occipital
mFus: middle Fusiform
mOTS: middle occipitotemporal sulcus
NHP: nonhuman primate
PAX6: paired box 6 gene
PCA: principal component analysis
pcw: postconception week
pFus: posterior fusiform
PHC: parahippocampal cortex
pOTS: posterior occipitotemporal sulcus
PPA: parahippocampal place area
pRF: population receptive field
RPKM: reads per kilobase million
TE: a high-level visual area in macaque inferotemporal cortex
TO: temporo-occipital
T1w: T1-weighted
T2w: T2-weighted
VO: ventral-occipital
VLTC: Ventrolateral Temporal Cortex
VTC: ventral temporal cortex
VWFA: visual word form area
V3AB: the combination of two visual areas (V3A and V3B) in human visual cortex located anterior to the dorsal portion of the third visual area
WPGMA: weighted-pair group method with arithmetic mean
