# Supplementary figures and images for: Human visual cortex is organized along two genetically opposed hierarchical gradients with unique developmental and evolutionary origins
Source: PLoS Biol. 2019 Jul 3;17(7):e3000362. doi: 10.1371/journal.pbio.3000362 (PMC6634416; doi:10.1371/journal.pbio.3000362)

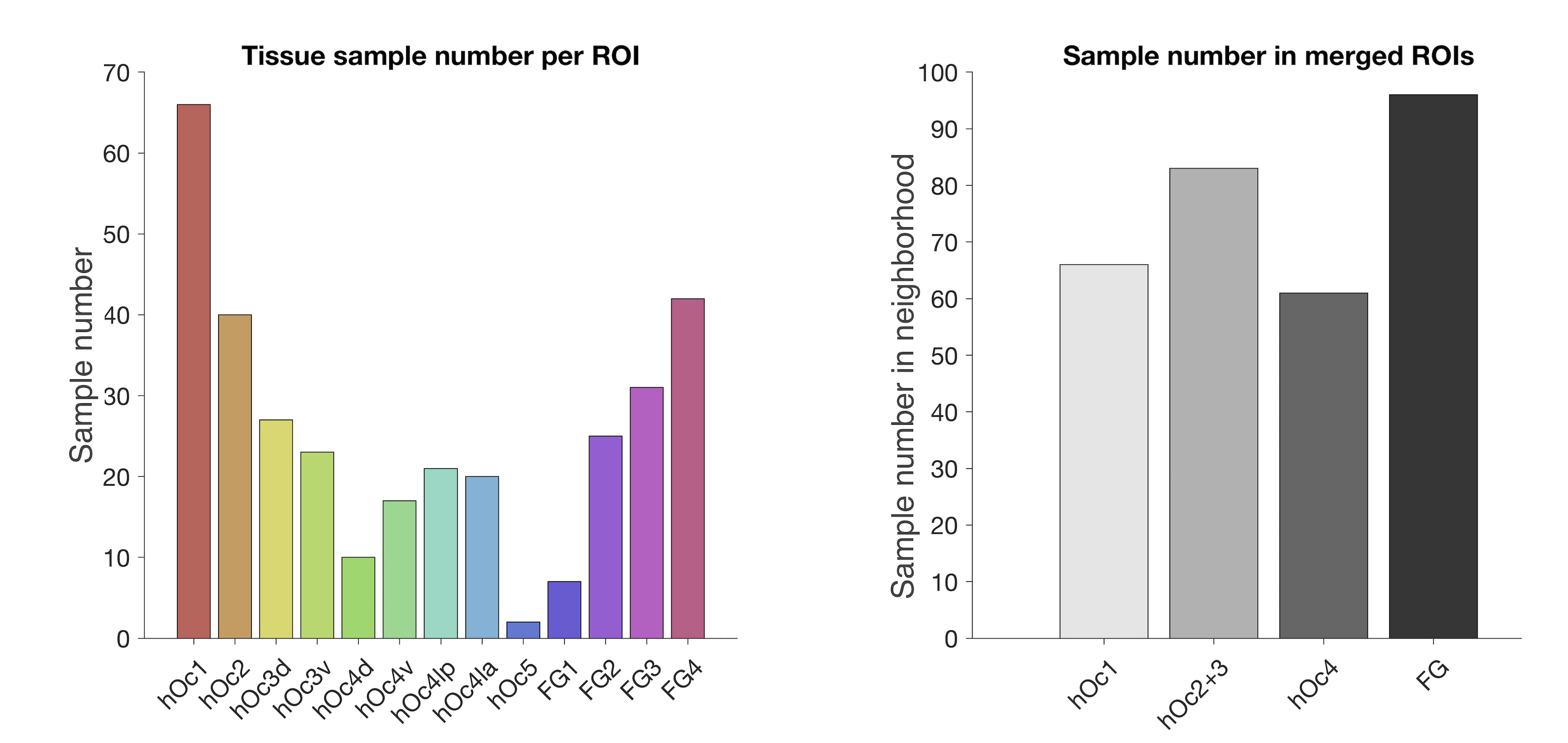

Supplement: S1 Fig — Left: once the cROIs were aligned to the transcriptomic data (Materials and Methods), we quantified the number of tissue samples from the 6 postmortem brains included in the AHBA that were located within each cROI. The colors for each cROI are the same as in Fig 1B. Right: prior to gene selection, we first grouped expression samples into four cytoarchitectonic neighborhoods (differently shaded gray bars) because there were an unequal number of tissue samples in each cROI. The hOc4 grouping includes samples from hOc4d, hOc4lp, hOc4la, and hOc4v. The FG grouping includes regions FG1 through FG4. Given the small number of tissue samples from hOc5 (n = 2), we excluded it in the final ANOVA to avoid bias. Importantly, the identification of the ascending and descending gene gradients is not dependent on the method used for gene selection (S2 Fig). AHBA, Allen Human Brain Atlas; ANOVA, analysis of variance; cROI, cytoarchitectonic region of interest; FG, fusiform gyrus; hOc, human occipital. (TIFF) [file pbio.3000362.s001.tiff]

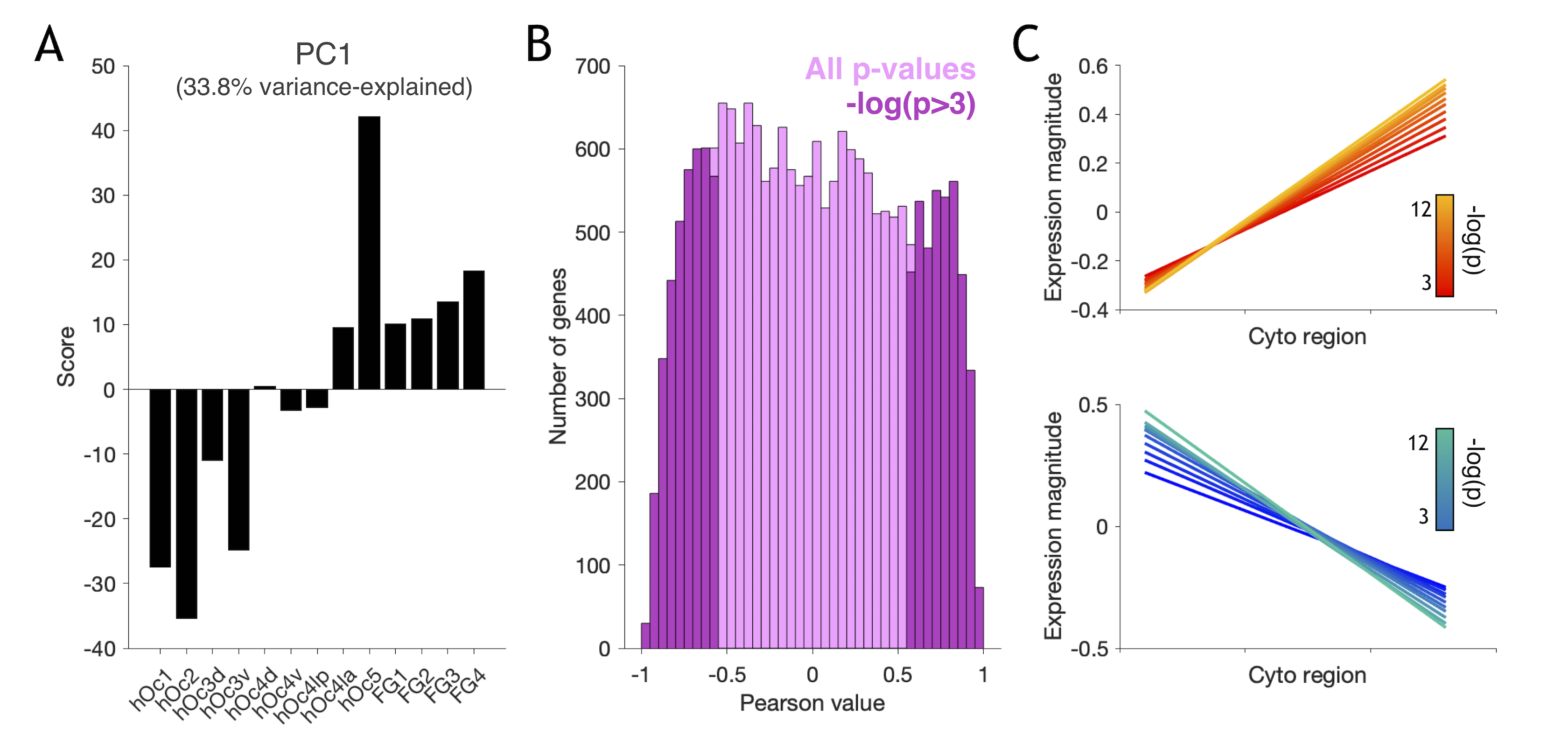

Supplement: S2 Fig — (A) We submitted the average expression magnitude of all genes within each of the 13 cROIs to PCA, the first component of which demonstrates a gradient of weighting scores, with posterior visual areas (e.g., hOc1, hOc2, etc.) mapping negatively onto this PC and anterior (e.g., FG2, FG3, etc.) visual regions mapping positively. (B) Histogram of Pearson correlation significance values (negative log-transformed) between each individual gene’s expression magnitude across the 13 cROIs with the scores of the first PC. Those genes with–log p-values exceeding 3 are highlighted in darker pink. (C) Linear fits summarizing the expression magnitude across cROIs of genes demonstrating either positive (warm colors, top) or negative (cool colors, bottom) correlations with PC1 scores. We took a stepwise approach, first including all genes whose–log p-values exceeded 3 in the average and then incrementally increasing the threshold until we only included genes in each group whose p-values exceeded 12, which included approximately 200 genes, equivalent to the group of 200 genes we chose in Fig 1. The magnitude of the positive or negative slope describing the expression gradients across cROIs increases as one includes more significantly differentially expressed genes. These analyses reveal that our results are not dependent on the method used to select the genes. With either method, two opposing gradients are identified. With the PCA approach, the two gradients represent positive and negative directions on the first PC, which explains 33.8% of the variance. cROI, cytoarchitectonic region of interest; FG, fusiform gyrus; hOc, human occipital; PCA, principal component analysis. (TIFF) [file pbio.3000362.s002.tiff]

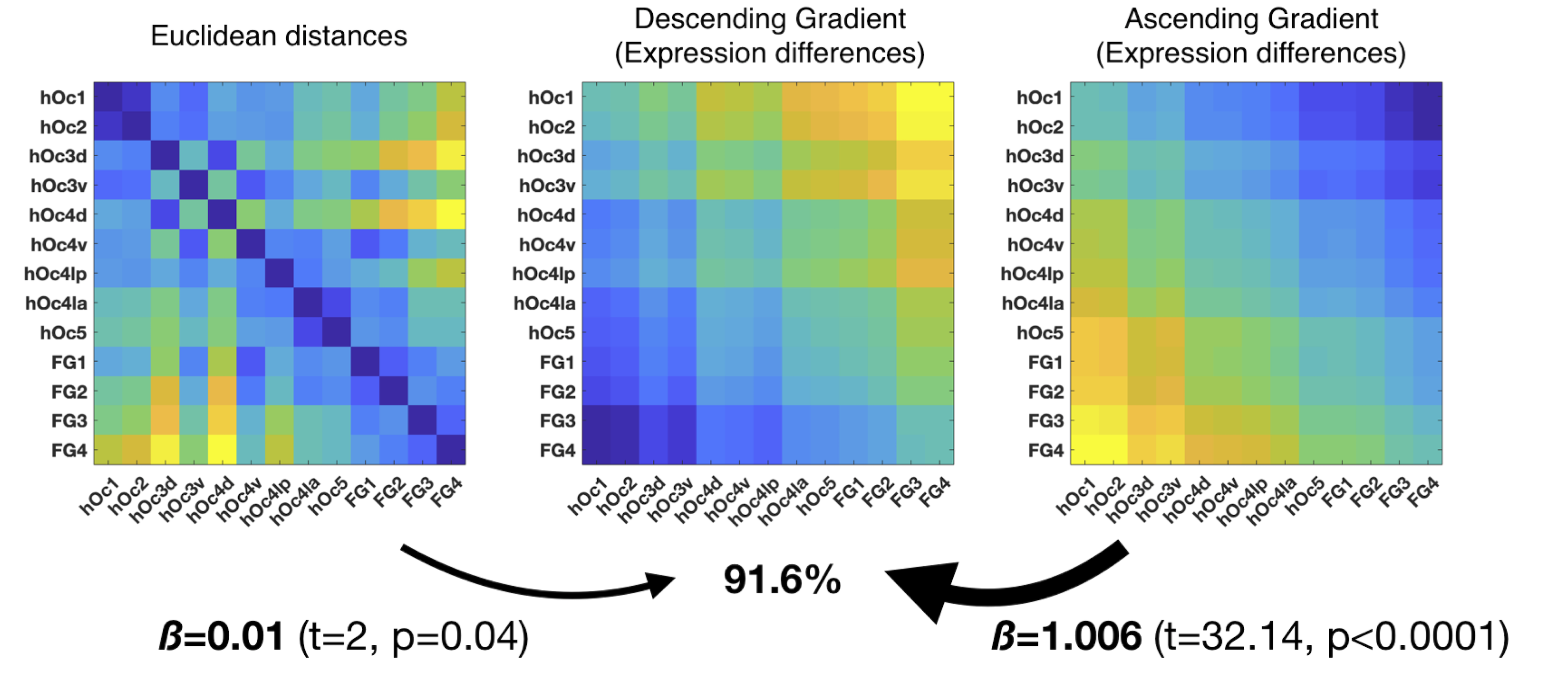

Supplement: S3 Fig — Left: inter-regional distances measured in MNI space between all cROIs of interest. Middle: inter-regional differences in the mean expression magnitude of descending-gradient genes. The descending-gradient matrix is the matrix whose values are being predicted by a stepwise regression, including the Euclidean distance and expression magnitude of ascending-gradient genes (right) as regressors. The beta-weights and significance of each regression are written beneath each matrix. cROI, cytoarchitectonic region of interest; MNI, Montreal Neurological Institute. (TIFF) [file pbio.3000362.s003.tiff]

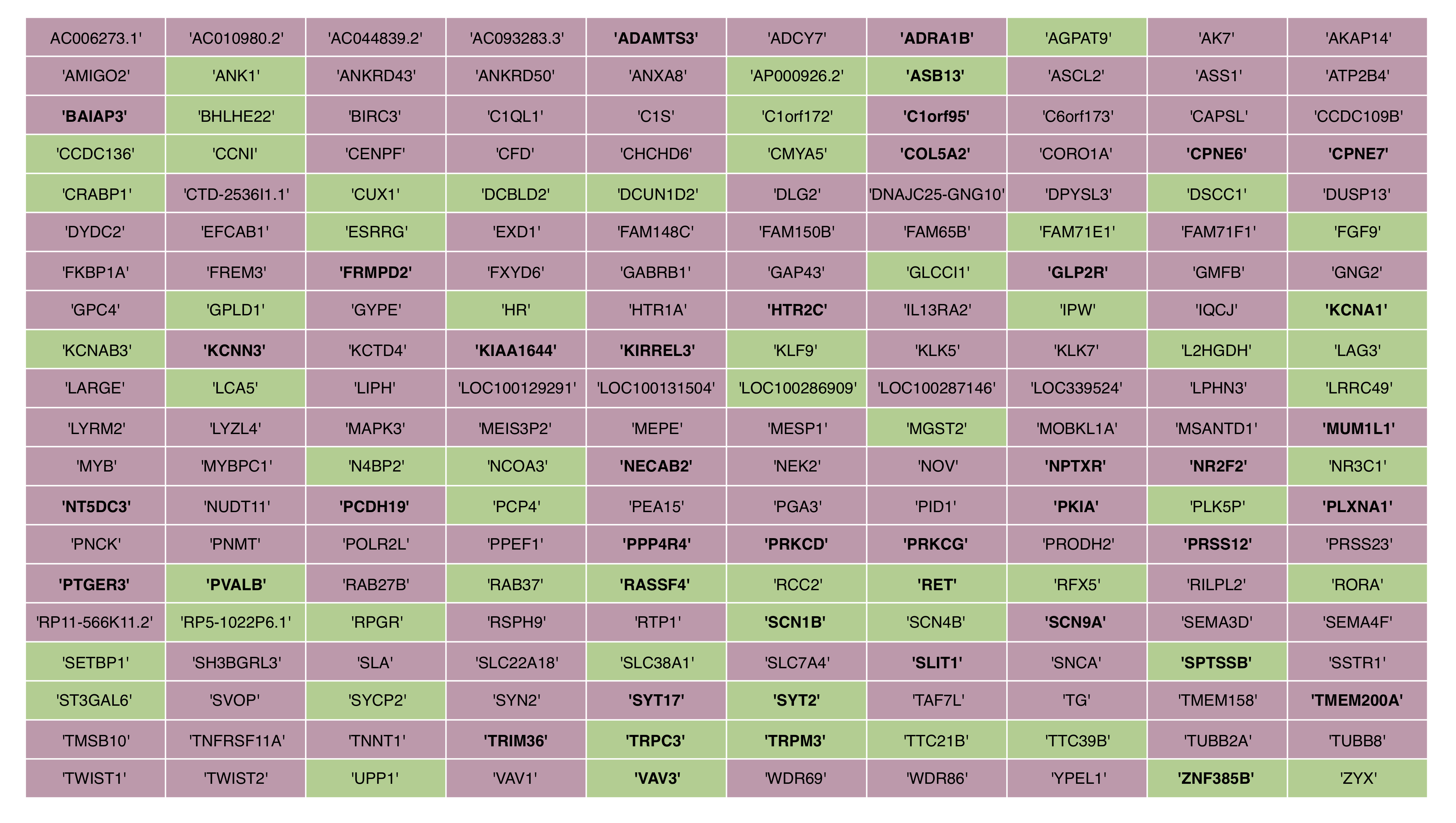

Supplement: S1 Table — Genes written in green belong to the descending gradient cluster, while those in pink belong to the ascending gradient cluster. Bolded gene names are those that were identified by Lake and colleagues [54] as differentiating specific neuronal subtypes. (TIFF) [file pbio.3000362.s004.tiff]

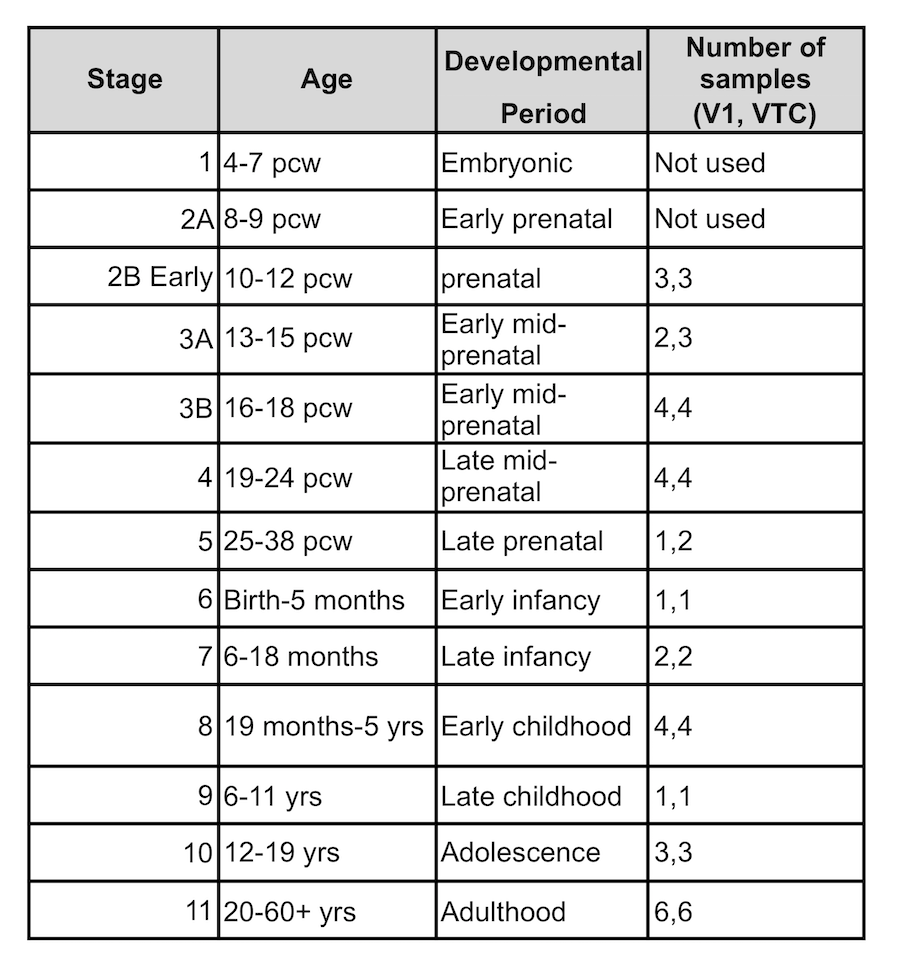

Supplement: S2 Table — This information and relevant data can be found at brainspan.org. pcw, postconception week. (TIFF) [file pbio.3000362.s005.tiff]
